# The *Escherichia coli* MarA protein regulates the *ycgZ*‐*ymgABC* operon to inhibit biofilm formation

**DOI:** 10.1111/mmi.14386

**Published:** 2019-09-29

**Authors:** Rachel A. Kettles, Natalia Tschowri, Kevin J. Lyons, Prateek Sharma, Regine Hengge, Mark A. Webber, David C. Grainger

**Affiliations:** ^1^ School of Biosciences Institute of Microbiology and Infection University of Birmingham Edgbaston Birmingham B15 2TT UK; ^2^ Institut für Biologie/Mikrobiologie Humboldt‐Universität zu Berlin 10115 Berlin Germany; ^3^ Quadram Institute Bioscience Norwich Research Park Norwich NR4 7UQ UK

## Abstract

The *Escherichia coli marRAB* operon is a paradigm for chromosomally encoded antibiotic resistance. The operon exerts its effect via an encoded transcription factor called MarA that modulates efflux pump and porin expression. In this work, we show that MarA is also a regulator of biofilm formation. Control is mediated by binding of MarA to the intergenic region upstream of the *ycgZ*‐*ymgABC* operon. The operon, known to influence the formation of curli fibres and colanic acid, is usually expressed during periods of starvation. Hence, the *ycgZ*‐*ymgABC* promoter is recognised by σ^38^ (RpoS)‐associated RNA polymerase (RNAP). Surprisingly, MarA does not influence σ^38^‐dependent transcription. Instead, MarA drives transcription by the housekeeping σ^70^‐associated RNAP. The effects of MarA on *ycgZ*‐*ymgABC* expression are coupled with biofilm formation by the *rcsCDB* phosphorelay system, with YcgZ, YmgA and YmgB forming a complex that directly interacts with the histidine kinase domain of RcsC.

## Graphical abstract

Expression of the multiple antibiotic resistance activator (MarA) protein can give rise to clinically relevant drug resistance in *Escherichia coli*. Counterintuitively, we showed that MarA production inhibits the formation of biofilms. Inhibition is mediated by the activation of the *ycgZ*‐*ymgABC* operon. To activate these genes, MarA acts selectively thus enhancing transcription by RNAP associated with the σ^70^ but not with the σ^38^ factor.

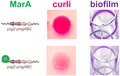

## Introduction

The *Escherichia coli* multiple antibiotic resistance (*mar*) locus was discovered as a genetic element providing resistance to tetracycline (George and Levy, 1983). The region encodes an operon designated *marRAB* and also provides resistance to quinolones, β‐lactams and a range of phenolic compounds (George and Levy, 1983; Ariza *et al.*, 1994; White *et al.*, 1997). Usually transcribed stochastically, constitutive *marRAB* expression can result from mutation (Cohen *et al.*, 1993; Ariza *et al.*, 1994; El‐Meouche *et al.*, 2016). Hence, clinical levels of drug resistance are associated with the inactivation of *marR* that encodes an auto repressor (Cohen *et al.*, 1993; Ariza *et al.*, 1994). Salicylic acid, and related phenolic molecules can also reduce repression by altering the conformation of MarR (Duval *et al.*, 2013; Hao *et al.*, 2014). The ability of the operon to provide resistance against antimicrobial compounds is dependent on *marA* that encodes a transcriptional activator (Ariza *et al.*, 1994; Rhee *et al.*, 1998). MarA plays an important role in drug resistance by activating the expression of the *acrAB*‐*tolC* encoded efflux pump (White *et al.*, 1997; Zhang *et al.*, 2008).

Many bacterial transcription factors act as dimers at palindromic DNA sequences (Robison *et al.*, 1998; Aravind *et al.*, 2005). In contrast, MarA binds to its DNA site, the marbox, as a monomer (Rhee *et al.*, 1998). Hence, MarA–DNA complexes are asymmetrical with defined orientation (Martin *et al.*, 1999). Promoters regulated by MarA can be divided into two classes. At class I promoters the marbox (5′‐GCAHWWWWTGYYAAA‐3′) is usually in the reverse orientation and located between ~50 and 70 base pairs (bp) upstream of the transcription start site (Martin *et al.*, 1999). Consequently, MarA contacts the RNA polymerase (RNAP) α subunit C‐terminal domain (αCTD) to activate transcription (Martin *et al.*, 1999). This interaction requires a surface of MarA comprising residues D18, W19, D22 and R36 (Dangi *et al.*, 2004). At class II promoters, the marbox is in the forward orientation and overlaps the promoter −35 element (Martin *et al.*, 1999). Hence, a contact with region 4 of the RNAP σ subunit may be involved (Zafar *et al.*, 2011). In recent work, we identified more than 30 transcription units directly targeted by MarA (Sharma *et al.*, 2017). A current aim is to understand the regulation and physiological functions of these targets.

Biofilms are populations of bacterial cells coalesced within a complex matrix of DNA, proteins and polysaccharides (Hall‐Stoodley *et al.*, 2004; Flemming, *et al.*, 2016). As well as being structural, the matrix helps to protect cells from damage (Hall‐Stoodley *et al.*, 2004; Flemming, *et al.*, 2016). Hence, biofilms may permit cell survival upon antibiotic treatment (Stewart and Costerton, 2001). In *E. coli*, the ability to form biofilms is regulated by the second messenger cyclic‐di‐GMP (Simm *et al.*, 2004). The downstream signalling pathway enhances the expression of a transcriptional activator called CsgD (Hammar *et al.*, 1995; Weber *et al.*, 2006). Subsequently, curli fibres are produced (Hammar *et al.*, 1995). These amyloid fibres facilitate surface adhesion, cell aggregation and are a major component of the biofilm matrix (Serra *et al.*, 2013; Hobley *et al.*, 2015). Curli expression can be inhibited by products of the *ycgZ*‐*ymgABC* operon. Briefly, these proteins induce the *rcsCDB*‐encoded phosphorelay system that reduces the levels of CsgD via the RprA sRNA (Tschowri *et al.*, 2009; Mika *et al.*, 2012; Tschowri *et al.*, 2012). In this work, we showed that, in addition to controlling the expression of efflux pumps, MarA directly activates the *ycgZ*‐*ymgABC* operon and so represses the formation of curli fibres and biofilms. Activation of *ycgZ*‐*ymgABC* proceeds via a class I mechanism whereby MarA binds to the 62 bp upstream of the *ycgZ*‐*ymgABC* promoter. Unusually, for class I promoters, the marbox is in the forward orientation and this is essential for activation. Stimulation of *ycgZ*‐*ymgABC* by MarA is σ factor specific. Hence, MarA drives transcription by RNAP associated with σ^70^ but not σ^38^. Consistent with regulation via the RcsCDB system, we show that *rcsB* is required for the effects of MarA on biofilm production mediated by *ycgZ*‐*ymgABC*. We also show that YcgZ, YmgA and YmgB form a complex that directly interacts with the histidine kinase (HK) domain of RcsC, presumably altering its phosphorylation state.

## Results

### MarA binds to a specific target site at the *ycgZ‐ymgABC* promoter

**Figure 1:**
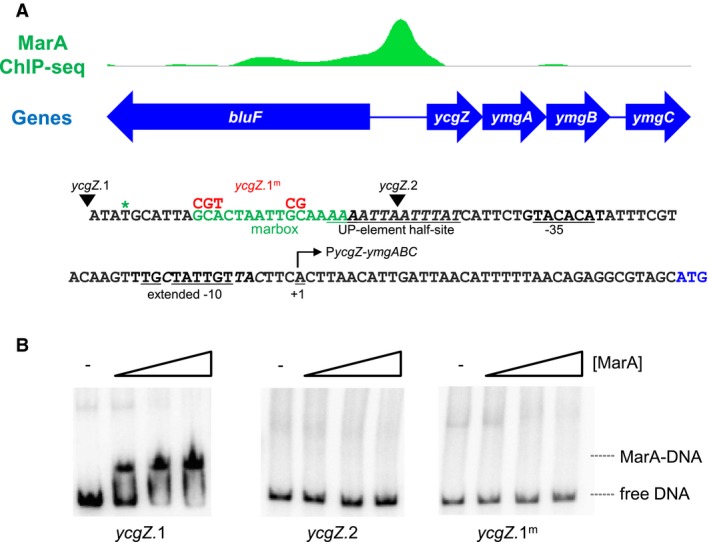
Binding of MarA to the *ycgZ‐ymgABC* intergenic region. A. ChIP‐seq data for MarA binding at the *ycgZ‐ymgABC* locus. Genes are shown as blue arrows and the ChIP‐seq data for MarA binding is in green (ArrayExpress accession number E‐MTAB‐5521). The sequence of the intergenic region, corresponding to the *ycgZ*.1 DNA fragment, is shown below the ChIP‐seq profile. The sequence of the predicted marbox is in green and the centre of the ChIP‐seq peak for MarA is denoted by an asterisk. The *ycgZ‐ymgABC* transcription start site is indicated by a bent arrow and the promoter extended −10 and −35 elements are underlined. Bases in italic are important for conferring recognition by σ^38^. Mutations introduced in the *ycgZ*.1^m^ DNA fragment are shown above the wild type DNA sequence in red. The 5' ends of the *ycgZ*.1 and *ycgZ*.2 DNA fragments are indicated by inverted triangles. B. Binding of MarA to the *ycgZ‐ymgABC* intergenic region *in vitro* requires the predicted marbox. The results of electrophoretic mobility shift assays are shown for different derivatives of the *ycgZ‐ymgABC* intergenic region. Where present, MarA was used at concentrations of 0.4, 1.2, or 2.0 μM. [Colour figure can be viewed at https://www.wileyonlinelibrary.com]

Previously, we used chromatin immunoprecipitation (ChIP) coupled with sequencing (ChIP‐seq) to map MarA binding across the *E. coli* genome (Sharma *et al.*, 2017). Locations bound by MarA included the intergenic region upstream of the *ycgZ*‐*ymgABC* operon. Figure 1A shows the ChIP‐seq data for MarA binding, the DNA sequence of the intergenic region and the predicted marbox. Our first aim was to determine if MarA bound at the proposed site. Hence, we generated a 119 bp DNA fragment corresponding to the sequence in Fig. 1A. This DNA fragment was named *ycgZ*.1. We also prepared mutated (*ycgZ*.1^m^) and truncated (*ycgZ*.2) derivatives. The mutations and site of truncation, both predicted to abolish MarA binding, are indicated alongside the *ycgZ*.1 DNA sequence in Fig. 1A. The ability of MarA to bind each of the DNA fragments was tested *in vitro* using electrophoretic mobility shift assays (Fig. 1B). As expected, MarA bound to the *ycgZ*.1 DNA fragment. However, MarA did not bind to *ycgZ*.1^m^ or *ycgZ*.2. Hence, MarA binds to the predicted site 62 bp upstream of the *ycgZ*‐*ymgABC* transcription start site.

### The *ycgZ‐ymgABC* promoter is recognised by σ^70^‐ and σ^38^‐associated RNA polymerase *in vitro*

**Figure 2:**
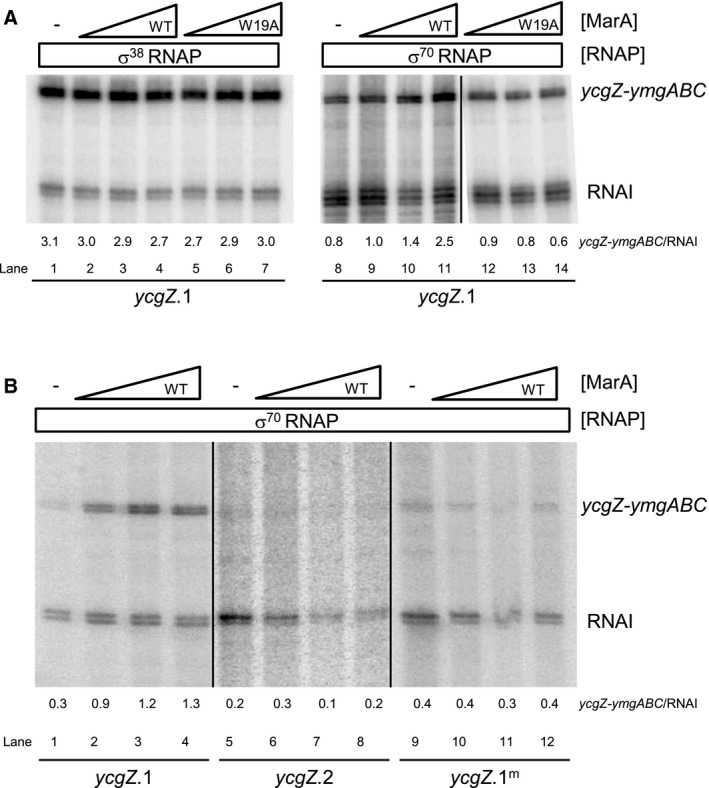
MarA activates transcription by σ^70^ but not σ^38^‐associated RNA polymerase at the *ycgZ‐ymgABC* promoter. A. Activation of the *ycgZ‐ymgABC* promoter requires MarA side chain W19. The figure shows *in vitro* transcription assays using the *ycgZ*.1 DNA fragment cloned in plasmid pSR as a template. RNA polymerase was used at a concentration of 0.4 μM and MarA was used at concentrations of 0.4, 1.2, or 2.0 μM. Transcripts generated from the *ycgZ‐ymgABC* promoter are labelled and the RNAI transcript is generated from the plasmid replication origin. B. Activation of the *ycgZ‐ymgABC* promoter requires the marbox. The figures show results of *in vitro* transcription assays. The DNA template was plasmid pSR carrying the *ycgZ*.1, *ycgZ*.1^m^ or *ycgZ*.2 DNA fragments. RNA polymerase was used at a concentration of 0.4 μM and MarA was used at concentrations of 0.4, 1.2 or 2.0 μM.

The *ycgZ‐ymgABC* operon is transcribed from a single promoter denoted P*ycgZ‐ymgABC* (Tschowri *et al.*, 2012) (Fig. 1A). Previous work noted reduced P*ycgZ‐ymgABC* activity in cells lacking σ^38^, the alternative RNAP sigma factor from starved cells (Tschowri *et al.*, 2009). Consistent with this, P*ycgZ‐ymgABC* exhibits features that specifically enhance σ^38^‐mediated transcription (bases italicised in Fig. 1) (Typas *et al.*, 2007a). We and others have previously shown that promoters recognised by σ^38^ can also be targets for the housekeeping σ^70^ factor (Typas *et al.*, 2007b; Grainger *et al.*, 2008; Singh *et al.*, 2011). Furthermore, even when utilising the same promoter, the two σ factors may respond differently to adjacently bound regulatory proteins (Colland *et al.*, 2000; Germer *et al.*, 2001; Grainger *et al.*, 2008; Singh *et al.*, 2011). To understand the ability of each RNAP derivative to utilise the *ycgZ‐ymgABC* promoter we used *in vitro* transcription assays. To facilitate this, the 119 bp *ycgZ*.1 DNA fragment was cloned in plasmid pSR upstream of the λ *oop* transcription termination signal. Hence, transcripts generated from the *ycgZ‐ymgABC* promoter are 128 nt in length and can be detected following electrophoresis. The result of the experiment is shown in Fig. 2A. The smaller RNAI transcript originates from the plasmid replication origin and serves as an internal control. As expected, σ^38^‐associated RNAP stimulated transcription from the *ycgZ‐ymgABC* promoter (lane 1). An identically sized transcript was produced by the σ^70^‐associated RNAP but with 4‐fold lower efficiency (lane 8). Hence, the *ycgZ‐ymgABC* promoter can be recognised by both RNAP derivatives.

### Binding of MarA at the *ycgZ‐ymgABC* promoter stimulates transcription by σ^70^‐associated RNA polymerase *in vitro*

We next sought to understand if MarA could alter transcription from the *ycgZ‐ymgABC* promoter. Hence, we added increasing concentrations of MarA to our *in vitro* transcription incubations. For reactions with σ^38^, there was no detectable change in transcription at any of the MarA concentrations tested (Fig. 2B, lanes 1–4). Conversely, σ^70^‐dependent transcription increased 3‐fold in the presence of MarA (Fig. 2B, lanes 8–11). In equivalent experiments, using the *ycgZ*.1^m^ or *ycgZ*.2 DNA sequences, the loss of the marbox prevented activation by MarA (Fig. 2B).

### Stimulation of σ^70^‐dependent transcription at the *ycgZ‐ymgABC* promoter requires MarA side chain W19

The position of the *ycgZ*‐*ymgABC* marbox suggests activation by a contact with the RNAP αCTD. Previously, Dangi and co‐workers (2004) identified a surface of MarA, including key amino acid residue W19, which mediates αCTD interactions. Hence, we purified MarA^W19A^ and tested its ability to stimulate transcription from P*ycgZ*‐*ymgABC*. The data are shown in Fig. 2A. As expected, there was no effect on the MarA independent transcription driven by σ^38^‐associated RNAP (lanes 5–7). Conversely, stimulation of σ^70^‐dependent transcription by MarA required residue W19 (compare lanes 8–11 with 12–14). Taken together, the position of the MarA binding site, and role of residue W19, are consistent with P*ycgZ*‐*ymgABC* stimulation involving a MarA contact with αCTD.

### Marbox position is important for σ^70^‐dependent activation of the *ycgZ‐ymgABC* promoter

**Figure 3:**
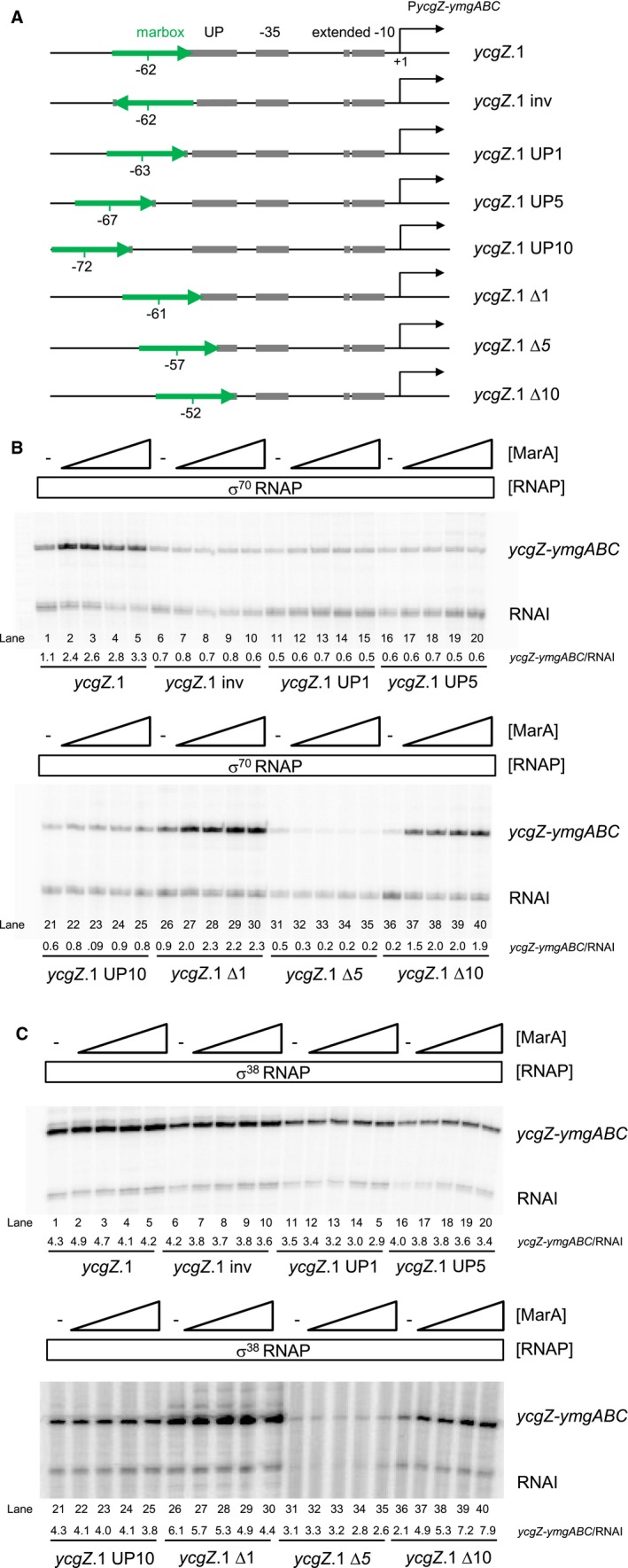
Spacing and orientation requirements for activation of the *ycgZ‐ymgABC* promoter by MarA. A. The schematics show the *ycgZ.*1 DNA fragment and derivatives. The marbox is shown as a green arrow to depict orientation. The centre of the marbox with respect to the transcription start site (black bent arrow) is indicated. Promoter elements are in grey and labelled. Note that the UP‐element half‐site is labelled ‘UP’ for brevity. B. Activation of transcription from different derivatives of *ycgZ*.1 by σ^70^‐associated RNA polymerase. The data are images of gels used to separate products from *in vitro* transcription assays. The DNA template was plasmid pSR carrying the different *ycgZ*.1 derivatives. RNA polymerase was used at a concentration of 0.4 μM and MarA was used at concentrations of 0.4, 0.8, 1.2, or 2.0 μM. C. Activation of transcription from different derivatives of *ycgZ*.1 by σ^38^‐associated RNA polymerase. Data are otherwise as described for panel B. [Colour figure can be viewed at https://www.wileyonlinelibrary.com]

The forward orientation of the marbox at the *ycgZ*‐*ymgABC* regulatory region is unexpected; all other class I MarA activated promoters contain a marbox in the reverse orientation (Martin *et al.*, 1999). The only exception is the *zwf* promoter where the marbox is positioned unusually close to the promoter −35 element (Martin *et al.*, 1999). Hence, we next sought to understand the importance of marbox orientation and position upstream of P*ycgZ*‐*ymgABC*. To do this, we created a series of *ycgZ*.1 derivatives in plasmid pSR. The full DNA sequences are shown in Fig. S1 and schematic illustrations are in Fig. 3A. In each case, either the position or orientation of the marbox was been altered (Fig. 3A). The consequences were measured using *in vitro* transcription assays (Fig. 3B). Whereas σ^70^‐dependent transcription from the starting *ycgZ*.1 fragment was enhanced by MarA (Fig. 3B, lanes 1–5), MarA could not stimulate transcription when the marbox was in the reverse orientation (lanes 6–10). Activation by MarA was also abolished when the marbox was moved upstream by 1 bp (lanes 11–15), 5 bp (lanes 16–20) or 10 bp (lanes 21–25). Positioning the marbox closer to the promoter was better tolerated. Thus, activation was observed when the marbox was −61 (lanes 26–30) or −52 (lanes 36–40) bp upstream of the transcription start site. Moving the marbox 5 bp closer to P*ycgZ*‐*ymgABC* was deleterious to both basal promoter activity and activation by MarA (lanes 31–35)*.*

### MarA activates σ^38^‐dependent transcription from a *ycgZ‐ymgABC* promoter derivative with a repositioned marbox

Interestingly, the *ycgZ*‐*ymgABC* promoter has a distal (i.e. not abutting the −35 hexamer) UP‐element half‐site (Fig. 1A). Previously, we showed that RNAP associated with σ^38^ is able to utilise such sequences. Conversely, σ^70^ containing holoenzyme is often defective at such promoters (Typas and Hengge, 2005). The P*ycgZ*‐*ymgABC* promoter derivatives described above have different UP‐element configurations (Fig. 3A). Hence, we also measured the ability of σ ^38^ bound RNAP to utilise the variants (Fig. 3C). Inverting or moving the marbox further upstream left the UP‐element half‐site intact. These changes had little impact on σ^38^‐dependent transcription (Fig. 3C, lanes 1–30). Moving the marbox 5 bp closer to the −35 element simultaneously deleted 5 bp of UP‐element DNA. This promoter derivative was poorly able to drive transcription by σ^38^ holoenzyme (lanes 31–35). Strikingly, when the marbox was positioned 10 bp further downstream, replacing the UP‐element half‐site, σ^38^‐dependent transcription was reduced (lane 36) but could be stimulated ~4‐fold by MarA (lanes 36–40).

### The *ycgZ‐ymgABC* promoter marbox is required for maximal activity in vivo

**Figure 4:**
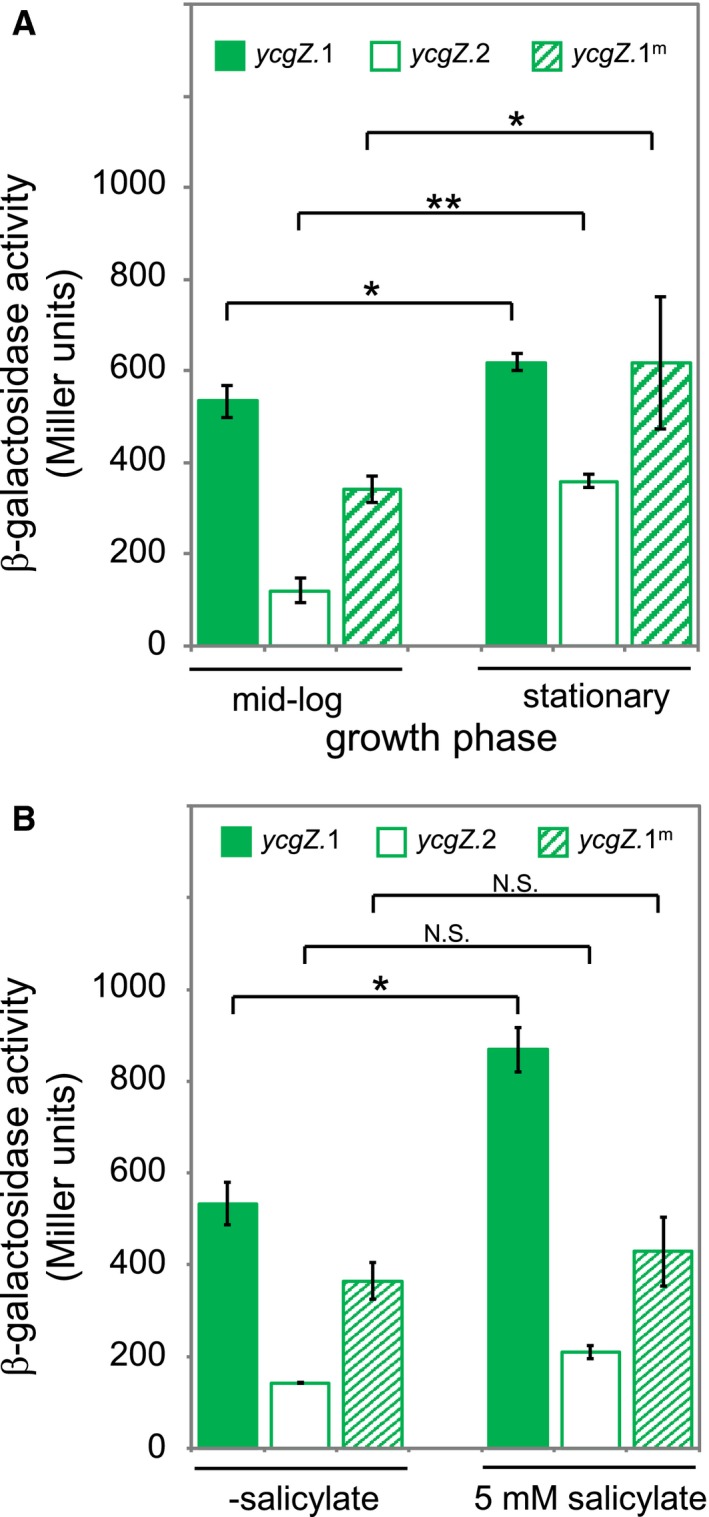
The *ycgZ‐ymgABC* promoter marbox is required for maximal activity *in vivo* during exponential growth and in the presence of salicylic acid. A. The graphs show levels of β‐galactosidase activity measured in lysates of *E. coli* strain JCB387 carrying different *ycgZ*::*lacZ* fusions in plasmid pRW50. Cultures in M9 minimal media were grown to exponential phase or stationary phase as indicated. The value of P was calculated using a two‐tailed student's *t*‐test. B. Levels of β‐galactosidase activity measured in lysates of cells grown in the presence of 5 mM sodium salicylate. The value of *P* was calculated using a two‐tailed student's *t*‐test. [Colour figure can be viewed at https://www.wileyonlinelibrary.com]

To understand the role that MarA might play in controlling *ycgZ‐ymgABC* transcription *in vivo* we fused various P*ycgZ‐ymgABC* DNA fragments to *lacZ* in the reporter plasmid pRW50. The plasmid constructs were used to transform *E. coli* Δ*lac* strain JCB387 and cells were grown to either mid‐log phase or stationary phase in Luria Broth. The cells were then lysed and β‐galactosidase activities determined using the lysates. The data are shown in Fig. 4A. In the presence of the marbox, β‐galactosidase activities were similar for growing and stationary phase cells (solid green bars). Deletion (open bars) or mutation (striped bars) of the marbox caused larger decreases in transcription for growing cells compared to starved cells (Fig. 4A). Recall that our *in vitro* transcription assays showed stimulation of σ^70^‐ but not σ^38^‐dependent transcription from P*ycgZ‐ymgABC* by MarA (Fig. 2A). Hence, the reduced requirement for the marbox in starved cells is probably due to an increase in σ^38^‐dependent transcription.

### The *ycgZ‐ymgABC* promoter marbox is required for induction by salicylic acid *in vivo*

Promoters activated by MarA can be induced with sodium salicylate (Duval *et al.*, 2013) because salicylic acid relieves repression of the *marRAB* operon by MarR (Duval *et al.*, 2013). We reasoned that transcription from the *ycgZ*‐*ymgABC* promoter should increase in the presence of salicylic acid. Furthermore, any such increase should require the marbox. Hence, we repeated our measurements of *ycgZ*‐*ymgABC* promoter activity in growing cells with or without the addition of 5 mM sodium salicylate (Fig. 4B). As expected, *ycgZ*‐*ymgABC* transcription increased upon the addition of sodium salicylate (compare solid bars). Conversely, little or no increase was observed when the marbox was deleted (open bars) or changed by mutation (striped bars).

### Curli fibre formation is inhibited by the *ycgZ‐ymgABC* operon in a marbox dependent manner

**Figure 5:**
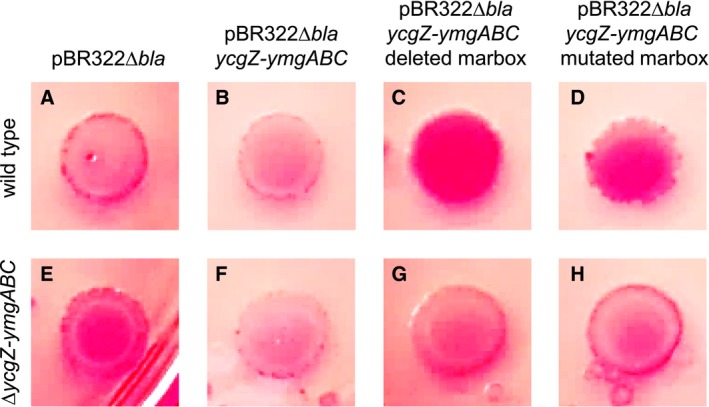
Expression of the *ycgZ‐ymgABC* operon reduces curli fibre production in a marbox dependent manner. Each of the panels (A) through (H) shows a macrocolony grown on Congo red agar plates. Images in panels A–D are of wildtype *E. coli* strain JCB387 transformed with empty plasmid vector (A), plasmid vector encoding *ycgZ‐ymgABC* under the control of its native promoter (B), or derivatives lacking (C) or having a mutated (D) *ycgZ‐ymgABC* marbox. Equivalent data are shown in panels E‐H for a JCB387 derived strain lacking the chromosomal *ycgZ‐ymgABC* operon. [Colour figure can be viewed at https://www.wileyonlinelibrary.com]

The *ycgZ*‐*ymgABC* operon inhibits the formation of biofilms by indirectly reducing the formation of curli fibres (Tschowri *et al.*, 2009; Mika *et al.*, 2012; Tschowri *et al.*, 2012). Briefly, expression of *ycgZ‐ymgABC* ultimately reduces the abundance of CsgD; a positive regulator of curli production. To understand the role of MarA, we made derivatives of plasmid pBR322Δ*bla*. These DNA constructs encoded *ycgZ‐ymgABC* under the control of its own promoter and the upstream marbox. We also made variants of the plasmid where the marbox was mutated or deleted as in Fig. 1A. The plasmids were used to transform *E. coli* JCB387 or a Δ*ycgZ*‐*ymgABC* derivative. Production of curli was then monitored in macrocolonies grown on agar plates containing Congo red dye that binds the fibres (Reichhardt *et al.*, 2015). Results are shown in Fig. 5. First, we compared macrocolonies formed by JCB387, or the Δ*ycgZ*‐*ymgABC* derivative, carrying the control pBR322Δ*bla* with no cloned insert. Wild type colonies had a pale pink appearance and a red ring at their periphery (panel A). Conversely, Δ*ycgZ*‐*ymgABC* colonies were red with a narrow pink ring just inside the border of the colony (panel E). As expected, the introduction of the plasmid encoding *ycgZ‐ymgABC*, under the control of P*ycgZ‐ymgABC* and the upstream marbox, reduced curli production. Hence, both wild type (panel B) and Δ*ycgZ*‐*ymgABC* (panel F) colonies had the same pale pink appearance. Removal or mutation of the plasmid marbox triggered an increase in curli production. In wild type cells, this was evident as a solid red macrocolony (panels C and D). Similarly, cells lacking Δ*ycgZ*‐*ymgABC* exhibited a red ring on the periphery of the colony and a deeper interior pink colour (panels G and H).

### Biofilm formation is inhibited by the *ycgZ‐ymgABC* operon in a marbox dependent manner

**Figure 6:**
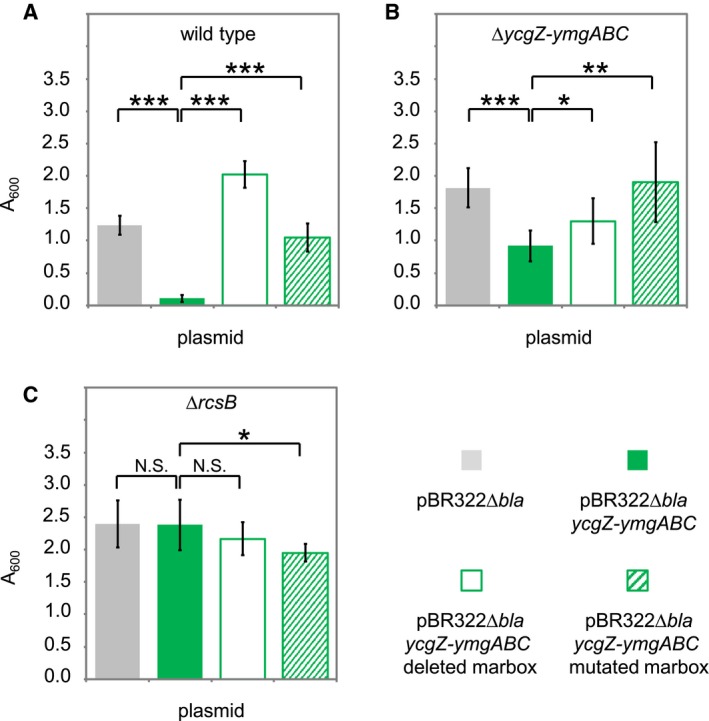
Expression of the *ycgZ‐ymgABC* operon reduces biofilm formation in a marbox and *rcsB* dependent manner. The figure shows results from assays of biofilm formation. Briefly, biofilms grown in culture plates were stained with crystal violet dye. After washing away excess dye, biofilms were dried and the dye solubilised. Spectrophotometry was then used to quantify the amount of dye bound by each biofilm. Data are shown for (A) wildtype *E. coli* strain JCB387 and derivatives lacking (B) *ycgZ‐ymgABC* or (C) *rcsB*. The strains were transformed with different plasmids. The plasmid derivatives are indicated by the key at the bottom of the figure. Each data point is the mean value from four independent biofilms and error bars indicate standard deviation. For each panel, the value of *P* was calculated using a two‐tailed student's *t*‐test. [Colour figure can be viewed at https://www.wileyonlinelibrary.com]

Biofilms are complex structures involving many extracellular components in addition to curli fibres (Tschowri *et al.*, 2009; Hobley *et al.*, 2015; Flemming *et al.*, 2016). Hence, we next investigated the role of *ycgZ*‐*ymgABC*, and upstream marbox, in controlling biofilm formation in cell culture plates. As described above, we tested different combinations of wild type *E. coli* JCB387, and the Δ*ycgZ*‐*ymgABC* derivative, carrying plasmid‐encoded *ycgZ*‐*ymgABC* with variants of the upstream regulatory DNA. Crystal violet dye was used to detect biofilms formed and the amount of dye bound by the biofilm was quantified by spectrophotometry. We first compared biofilms formed by JCB387, or the Δ*ycgZ*‐*ymgABC* derivative, carrying the control pBR322Δ*bla* with no cloned insert (Fig. 6A and 6B, grey bars). There was a small increase in the Δ*ycgZ*‐*ymgABC* strain (*P* = 1.1e−5). As expected, the introduction of the plasmid encoding *ycgZ‐ymgABC*, under the control of P*ycgZ‐ymgABC* and the upstream marbox, reduced biofilm formation (Fig. 6A and 6B, green bars). Removal or mutation of the marbox triggered an increase in biofilm production (Fig. 6A and 6B, open and striped bars). Surprisingly, differences were most pronounced for the wild type JCB387 strain (Fig. 6A). We speculate that deleting chromosomal *ycgZ*‐*ymgABC* may have additional uncharacterised downstream consequences.

### Regulation of *ycgZ‐ymgABC* by MarA is uncoupled from biofilm formation in cells lacking rcsB

Recall that the *ycgZ*‐*ymgABC* operon exerts its effect on biofilms by activating the RcsCDB phosphorelay system (Tschowri *et al.*, 2009; Mika *et al.*, 2012; Tschowri *et al.*, 2012). Briefly, RcsC is an inner membrane sensor kinase that can phosphorylate the phosphotransferase RcsD. In turn, RcsD phosphorylates the response regulator RcsB that activates the expression of a sRNA called RprA. The sRNA inhibits the translation of CsgD; a positive regulator of curli production and biofilm formation (Tschowri *et al.*, 2009; Mika *et al.*, 2012). Hence, we reasoned that effects of MarA on biofilms, mediated by *ycgZ*‐*ymgABC*, should be abolished in cells lacking RcsB. To test this prediction, we repeated our assays of biofilm production in derivatives of the *E. coli* JCB387 strain lacking *rcsB*. As expected, deletion of *rcsB* increased the production of biofilms twofold (compare grey bars in Fig. 6A and 6C). In this genetic background introducing the plasmid encoding *ycgZ‐ymgABC*, under the control of P*ycgZ‐ymgABC* and the upstream marbox, had no effect (Fig. 6C, green, open and striped bars).

### Putative interactions between *ycgZ‐ymgABC* and rcsCDB‐encoded proteins are revealed by two‐hybrid analysis

**Figure 7:**
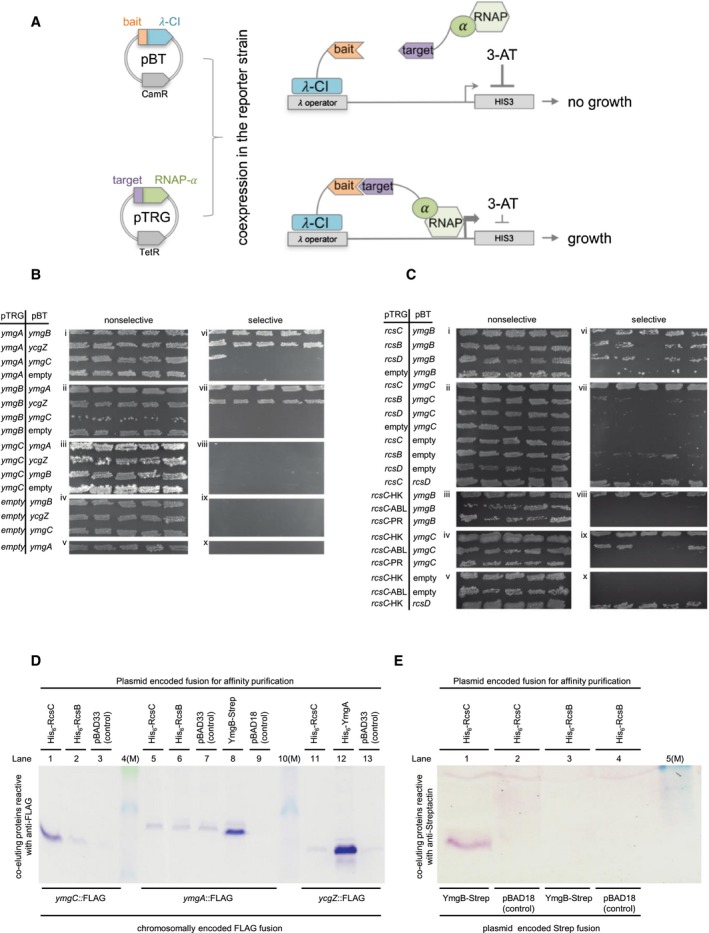
A complex formed by YmgA, YmgB and YcgZ interacts with RcsC. A. Schematic representation of the BacterioMatch two‐hybrid system. Plasmids pBT and pTRG are used to fuse prey proteins to λCI or replace the RNA polymerase α C‐terminal domain with target proteins. Upon expression *in vivo*, interactions between λCI‐prey and α‐bait fusions allow sufficient expression of His3 for growth in the presence of the His3 inhibitor 3‐AT. B. BacterioMatch two‐hybrid system reporter cells were cotransformed with derivatives of the pBT and pTRG plasmids and vector‐only controls. YcgZ, YmgA, YmgB and YmgC were expressed either as hybrid proteins fused to cI‐NTD from pBT or as fusions to RNAP alpha‐NTD from pTRG. For experiments with RcsC or RcsD we cloned the entire cytosolic part of the protein or the indicated histidine kinase (RcsC‐HK), alpha‐beta‐loop (RcsC‐ABL) and phosphoreceiver domain (RcsC‐PR). Full‐size RcsB was expressed from pTRG. Interactions were detected by growth in the presence of the His3 inhibitor 3‐AT (selective) at 37°C for 24 h following incubation at 28°C for 48 h. Each row on the plates shows patches of five independent cotransformants. C. The *E. coli* strain MC4100 encoding chromosomal *ymgC*::Flag, *ymgA*::Flag or *ycgZ*::Flag was transformed with pBAD33‐derivatives expressing His_6_‐RcsC, His_6_‐RcsB, or His_6_‐YmgA. YmgB‐Strep was expressed from pBAD18 and the empty vectors served as negative controls. Flag‐tagged proteins coeluting in Ni‐NTA or Strep‐Tactin Sepharose based chromatography of cellular lysates were detected using immunoblot analysis and the monoclonal anti‐Flag antibody. D. Wild‐type MC4100 was cotransformed with pBAD18 or pBAD18‐*ymgB*‐Strep and either pBAD33‐His‐*rcsC* (cytosolic part) or pBAD33‐His‐*rcsB*. YmgB‐Strep coeluting with His‐RcsC or His‐RcsB from extracts subject to Ni‐NTA chromatography was detected using the anti‐Strep‐Tactin antibody. Lanes labelled (M) contain size markers. [Colour figure can be viewed at https://www.wileyonlinelibrary.com]

We next aimed to better understand how the *ycgZ*‐*ymgABC* gene products impact the RcsCDB phosphorelay system. In particular, we wondered if direct protein–protein interactions were involved. To test this, we utilised the BacterioMatch two‐hybrid system (Fig. 7A, Dove and Hochschild, 2004). The assay detects interactions between ‘bait’ and ‘target’ proteins fused to the λcI transcription factor and RNAP α subunit respectively. If fused proteins interact, the λcI derivative recruits modified RNAP to a semisynthetic promoter. This allows expression of the downstream yeast *HIS3* gene, required for histidine biosynthesis. Hence, interactions between ‘bait’ and ‘target’ allow *E. coli* to grow on media containing 3‐amino‐1,2,4‐triazole (3‐AT), an inhibitor of histidine production (Dove and Hochschild, 2004). Figure 7B shows the growth of *E. coli* harbouring different combinations of YcgZ, YmgA, YmgB and YmgC, fused to λcI or RNAP αNTD, in plasmids pBT and pTRG respectively. To check reproducibility, five individual colonies of each strain were ‘patched’ on both selective (with 3‐AT) and nonselective (no 3‐AT) media. As expected, cells were able to grow on nonselective media regardless of the plasmid combination used (Fig. 7B, i–v). Conversely, growth on selective media was only permitted by certain combinations of the various fusions. (Fig. 7B, vi–x). Specifically, all combinations of fusions containing YcgZ, YmgA and YmgB allowed growth on selective plates (Fig. 7B, vi–vii, top two rows). Hence, these three factors can all interact and might form a complex. In contrast, fusions with YmgC did not reproducibly stimulate growth in any combination (Fig. 7B, viii).

Next, we examined interactions between *ycgZ*‐*ymgABC*‐ and *rcsCDB*‐encoded proteins. We did not detect any interactions involving YcgZ or YmgA (data not shown). Conversely, a reproducible interaction was detected between YmgB and RcsC (Fig. 7C, vi, top row). For YmgB in combination with RcsB or RcsD, the data were erratic; growth was sparse and inconsistent (Fig. 7C, vi, middle two rows). Such ambiguities are not unusual and suggest a weak or artefactual interaction barely sufficient to permit survival (Tschowri *et al.*, 2012). A reproducible interaction was detected between YmgC and RcsC (Fig. 7C, vii, top row). We were also able to detect interactions between RcsC and RcsD (Fig. 7C, vii, bottom row). To better define interactions, we tested the ability of YmgB or YmgC to interact with individual RcsC domains. Hence, we cloned the HK, alpha‐beta‐loop (ABL) or phosphoreceiver (PR) domains of RcsC in pTRG. The data show that both YmgB and YmgC can contact the cytoplasmic HK domain of RcsC (Fig. 7C, top row of panels viii and ix). We were unable to obtain a reproducible result for the interaction of YmgC and RcsC‐ABL (Fig. 7C, ix, second row).

### Validation of protein–protein interactions by affinity purification and coelution

The two‐hybrid analysis suggests that YmgA, YmgB and YcgZ can all interact with each other (Fig. 7B). Furthermore, both YmgB and YmgC interact with RcsC (Fig. 7C). To independently validate these interactions we used *in vivo* coelution assays. Hence, we constructed plasmids encoding RcsC, RcsB, YmgA or YmgB with either a His_6_‐ or Strep‐tag. The plasmids were used to transform strains expressing Flag‐tagged YmgC, YmgA or YcgZ. After cell lysis, His_6_‐ or Strep‐tagged proteins were purified by affinity chromatography. Copurification of FLAG‐tagged proteins was probed by western blotting. The data show copurification of YmgC‐FLAG with His_6_‐RcsC (Fig. 7D, lane 1), YmgA‐FLAG with YmgB‐Strep (lane 8) and YcgZ‐Flag with His_6_‐YmgA (lane 12). To check interactions between YmgB and RcsB or RcsC we coexpressed YmgB‐Strep with His_6_‐ RcsB or RcsC. The His_6_ proteins were purified from cell lysates and the presence of YmgB‐Strep probed by western blotting. The YmgB‐Strep copurified with His‐RcsC (Fig. 7E, lane 1) but not RcsB (lane 3).

## Discussion

**Figure 8:**
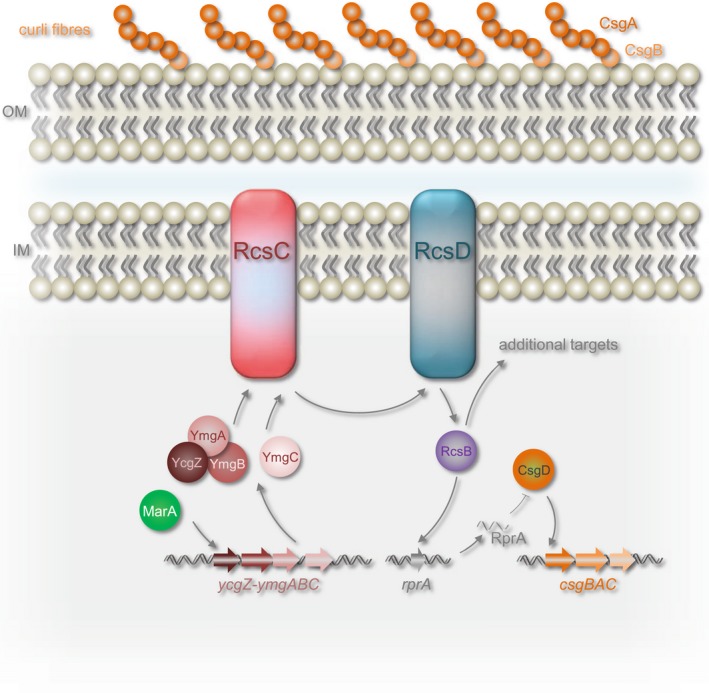
Model for repression of biofilm formation by MarA. Genes are shown as block arrows and proteins are shown as spheres or ovals. Stimulatory and inhibitory interactions are shown by arrows and bar‐headed lines respectively. Nucleic acids are shown as double (DNA) or single (sRNA) wavy lines. The inner membrane (IM), outer membrane (OM) and curli fibres are labelled. [Colour figure can be viewed at https://www.wileyonlinelibrary.com]

In this work, we show that MarA is a positive regulator of the *ycgZ*‐*ymgABC* promoter in *E. coli* (Figs 2 and 3). We also demonstrate that activation of *ycgZ*‐*ymgABC* by MarA reduces biofilm production in a manner requiring *rcsB* (Fig. 6). Hence, the simplest explanation is that MarA exerts its effect via the known ability of *ycgZ*‐*ymgABC* to stimulate the RcsCDB phosphorelay system. We show that *ycgZ*‐*ymgABC* targets RcsCDB directly; YmgB forms a complex with YcgZ and YmgA that contacts the HK domain of RcsC (Fig. 7). Since activation of the RcsCDB system triggers the production of the RprA sRNA, which inhibits CsgD expression, the production of curli fibres is reduced (Fig. 5) (Tschowri *et al.*, 2009; Mika *et al.*, 2012). Our model is summarised in Fig. 8. Note that the regulation of *ycgZ*‐*ymgABC* likely impacts other aspects of biofilm formation beyond curli production. For instance, the *bdm* (biofilm‐dependent modulation) gene is also subjected to regulation by the RcsCDB cascade (Francez‐Charlot *et al.*, 2005). It is initially counterintuitive that increased MarA production should inhibit biofilm formation; the biofilm mode of life is considered favourable for surviving treatment with antibiotics (Stewart and Costerton, 2001; Hall‐Stoodley *et al.*, 2004; Hobley *et al.*, 2015; Flemming *et al.*, 2016). However, for growing planktonic cells, 24 h are required to establish a biofilm (Elvers *et al.*, 2002; Adamus‐Białek *et al.*, 2015). Clearly, a biofilm must already exist to provide protection (Stewart and Costerton, 2001; Hall‐Stoodley *et al.*, 2004; Hobley *et al.*, 2015; Flemming *et al.*, 2016). Hence, nascent biofilm formation seems to be a poor strategy for surviving immediate threats. We suggest that, during planktonic growth, induction of the *mar* response inhibits biofilm formation and favours short term survival strategies including drug efflux, altered outer membrane permeability and DNA repair (White *et al.*, 1997; Sharma *et al.*, 2017).

Activation of the *ycgZ*‐*ymgABC* promoter requires MarA residue W19 (Fig. 2). Furthermore, MarA can exert its effect from different positions (Fig. 3). This suggests a contact with the RNAP αCTD (Gaston *et al.*, 1990; Wing *et al.*, 1995; Dangi *et al.*, 2004). Surprisingly, activation only occurs when RNAP is associated with the σ^70^ subunit; σ^38^‐dependent transcription from P*ycgZ‐ymgABC* is not stimulated by MarA. Hence, activation by MarA is sigma factor specific but does not require a direct interaction with σ^70^. It is well established that σ^70^ and σ^38^ have vastly different DNA bending capacities (Shin *et al.*, 2005). Furthermore, we have shown previously that the holoenzyme variants interact with UP‐elements differently; complexes with σ^38^ preferentially utilise promoter distal UP‐element half‐sites. At P*ycgZ‐ymgABC*, one such element is amongst several sequence characteristics that favour basal transcription initiation involving σ^38^ (Fig. 1A). The −13C base is directly contacted by σ^38^ K173 whilst nonoptimal spacers and AT‐rich discriminators are better tolerated by σ^38^ (Becker and Hengge‐Aronis, 2001; Typas and Hengge, 2006; Typas *et al.*, 2007a). The different interactions that αCTD makes in the context of σ^38^ holoenzyme also explain selective activation by MarA. Hence, the σ^38^ derivative preferentially uses the distal UP‐element half‐site whilst MarA provides a point of contact for αCTD in the context of σ^70^ holoenzyme. Consistent with this, moving the marbox 10 bp downstream, to replace the UP‐element, negates basal preference of the promoter for σ^38^ and permits MarA to activate σ^38^‐dependent transcription. To our knowledge, selective regulation has not previously been demonstrated for AraC family regulators. However, other transcription factors are known to behave in this way (Colland *et al.*, 2000; Germer *et al.*, 2001; Typa *et al.*, 2007b; Grainger *et al.*, 2008; Singh *et al.*, 2011). For example, OxyR, Fis and H‐NS regulate σ^70^ but not σ^38^‐dependent transcription at the *dps* locus (Grainger *et al.*, 2008). Similarly, at the *pro*P2 promoter, Fis activates σ^38^‐ but not σ^70^‐dependent transcription (Typas *et al.*, 2007b). Hence, P*ycgZ‐ymgABC* fits the general rule that σ^38^ can act more autonomously than σ^70^ (Typas *et al.*, 2007a).

Interestingly, Vila and Soto previously noted that MarA could inhibit biofilm formation in uropathogenic *E. coli* (UPEC) (Vila and Soto, 2012). It was suggested that the reduced production of type I fimbriae was responsible and that this was mediated via repression of *fimB* by MarA (Vila and Soto, 2012) We suggest that any such repression must be indirect since the *fimB* promoter does not contain a marbox (Sharma *et al.*, 2017). Conversely, UPEC strains do encode the *ycgZ*‐*ymgABC* operon and the position and sequence of the MarA binding site are conserved. Hence, an alternative explanation is that MarA repression of biofilm formation in UPEC involves the mechanism outlined here. We speculate that production of type I fimbriae may be modulated in response to expression of the *ycgZ*‐*ymgABC* operon (Fig. 8). We also note that Duval and coworkers reported the accumulation of *ycgZ*‐*ymgABC*‐encoded proteins in strains lacking the Lon protease (Duval *et al.*, 2017). This is due to Lon targeting MarA for degradation and levels of MarA thus increasing substantially in Lon deficient cells (Martin *et al.*, 2008). Hence, our work is concordant with, and provides an explanation for, several previous observations linking MarA, biofilm formation and control of *ycgZ*‐*ymgABC*.

## Experimental procedures

### Strains, plasmids and oligonucleotides

Strains and plasmids are listed in Table 1 and oligonucleotides in Table S1. To construct pET28a‐MarA, a DNA fragment encoding *marA* was generated by PCR using the MarA‐OE‐F and Mar‐OE‐R oligonucleotides. Following digestion with *Bam*HI and *Nde*I, the DNA was ligated downstream of the T7‐lac promoter in pET28a. The pBR322Δ*bla* plasmid was made by the digestion of pBR322 with *Aat*II and *Vsp*I to excise the β‐lactamase gene. A small linker with terminal *Aat*II and *Vsp*I sites was used to recircularise the plasmid. Fragments encoding *ycgZ‐ymgABC* operon, with variants of the upstream DNA, were then cloned in pBR322Δ*bla* via the *Eco*RI and *Aat*II restriction sites. The Δ*ycgZ‐ymgABC* derivative of *E. coli* strain JCB387 was created using the gene doctoring method described by Lee *et al. *(2009). We transferred *rcsB::*kan by P1 transduction from a derivative of strain BW25113 (Baba *et al.*, 2006). C‐terminally 3x Flag‐tagged chromosomally encoded *ymgC*::Flag, *ymgA*::Flag and *ycgZ*::Flag were constructed in *E. coli* K‐12 strain MC4100 using pSUB11 (Uzzau *et al.*, 2001) as a template for PCR and oligonucleotides listed in Table S1 following λRED‐based recombination procedure (Uzzau *et al.*, 2001). For *in vivo* interaction assays, BacterioMatch II Two‐Hybrid System vectors pBT and pTRG were used (Stratagene, Agilent Technologies). The relevant genes were cloned using primers listed in Table S1 to generate C‐terminal fusions either to the lambda cI repressor from pBT or to the N‐terminal domain of the α subunit of *E. coli* RNAP from pTRG and fusion proteins were tested for interaction in histidine auxotrophic XL1‐Blue MRF’‐derivative *E. coli* strain (Stratagene, Agilent Technologies). For *in vivo* coelution experiments, the soluble cytosolic parts of RcsC, RcsB and YmgA, respectively were N‐terminally fused to a 6xHis‐tag and cloned into pBAD33. YmgB carrying a Strep‐tag at the C‐terminus was cloned into pBAD18. pBAD33‐derived RcsC (cytosolic moiety only), RcsB and YmgA were either expressed in strains containing *ymgC*::Flag, *ymgA*::Flag or *ycgZ*::Flag, respectively, or together with cotransformed pBAD18‐*ymgB*‐Strep in MC4100. Coelution experiments were done with cell lysates as described further below.

**Table 1:** Strains and plasmids.

Name	Description	Source
*Bacterial strains*		
JCB387	Δ*nirB,* Δ*lac*	Page *et al. *(1990)
JCB387Δ*ycgZ‐ymgABC* Δ*rcsB*	Δ*nirB,* Δ*lac,* Δ*ycgZ‐ymgABC,* Δ*rcsB*	This work
JCB387Δ*ycgZ‐ymgABC* Δ*rcsB*	Δ*nirB,* Δ*lac,* Δ*ycgZ‐ymgABC,* Δ*rcsB*	This work
BL21 DE3	T7 RNApol + F‐ *ompT* rb‐ma‐ *fhuA2*	Studier (1991)
T7 Express	*lacZ*::T7 gene1 [*lon*] *ompT gal sulA*11	NEB
	R(*mcr*‐73::miniTn10‐‐TetS)2 [*dcm*]	
	R(*zgb*‐210::Tn10‐‐TetS) *endA*1	
	Δ(*mcrC*‐*mrr*)114::IS10	
MC4100	*E.coli* K12 F‐ *araD*139 O(*argF‐lac*)U169	Casadaban (1976)
	*deoC flbB*5301 *relA*1 *rpsL*150 *ptsF*25 *rbsR*	
NAT239	MC4100 *ycgZ::*Flag	This work
NAT240	MC4100 *ymgA::*Flag	This work
NAT242	MC4100 *ymgC::*Flag	This work
XL1‐Blue MRF	Δ(*mcrA*)183 Δ(*mcrCB*‐*hsdSMR*‐*mrr*)173	Agilent Technol.
	*endA*1 *supE*44 *thi*‐1 *recA*1 *gyrA*96	
	*relA*1 *lac* [F´ *proAB lacIq*ZΔM15 Tn5 (Kan^r^)]	
*Plasmids*		
pRW50	Low copy number 16 kb plasmid for making *lacZ* fusions. Contains the RK2 origin of replication and encodes TetR	Lodge *et al. *(1992)
pSR	4 kb pBR322 derivative that encodes AmpR. Contains an *Eco*RI‐*Hin*dIII cloning site upstream of the λ*oop* transcription terminator	Kolb *et al. *(1995)
pBR322	4.4kb, encodes TetR and AmpR. Contains the pMB1 origin of replication and *rop* for restriction of plasmid copy number	Bolivar *et al. *(1977)
pBR322Δ*bla*	pBR322 lacking the *bla* gene	This work
pET‐28a	5.4kb, encodes KanR. Contains the T7 *lac* promoter for high‐level IPTG‐inducible expression of recombinant proteins with N‐ or C‐terminal His tags and a thrombin cleavage site	Novagen
pDOC‐C	5.8kb, encodes AmpR, derived from pEX100T. Used as a cloning vector for gene doctoring. Features a cloning region flanked by two I‐SceI recognition sites	Lee *et al. *(2009)
pDOC‐K	Derived from pEX100T and contains a kanamycin resistance cassette between two Flp recombinase recognition sites	Lee *et al. *(2009)
pACBSR	7.3kb, encodes CamR. Recombination plasmid for gene doctoring; carries arabinose inducible λ‐Red and I‐SceI endonuclease genes	Herring *et al. *(2003)
pCP20	9.4kb, encodes CamR and AmpR. Encodes yeast FLP recombinase gene. Used to remove the kanamycin cassette in gene doctoring	Cherepanov and Wackernagel (1995)
pSUB11	3.5kb, encodes KanR. Used to amplify Flp recombinant target (FRT)‐flanked kanamycin resistance cassette with 3xFlag	Uzzau *et al.*, 2001
pKD46	6.3kb, encodes AmpR. Used to express λRed recombinase	Uzzau *et al.*, 2001
pBT	3.2kb, encodes CamR. BacterioMatch II Two‐Hybrid System vector. Encodes λ phage cI protein	Agilent Technol.
pTRG	4.4kb, encodes TetR. BacterioMatch II Two‐Hybrid System vector. Encodes RNAP αNTD	Agilent Technol.
pBAD18	4.6kb, encodes AmpR. Carries arabinose‐inducible *araBAD* promoter, pBR322 origin	Guzman *et al. *(1995)
pBAD33	5.3kb, encodes CamR. Carries arabinose‐inducible *araBAD* promoter, pACYC184 origin	Guzman *et al. *(1995)

### Protein purification

Preparations of σ^70^ and σ^38^ were made by the overexpression of the cloned *rpoD* and *rpoS* genes in BL21 DE3 cells, and subsequent purification by liquid chromatography, as described by Grainger *et al. *(2008). The RNAP core enzyme was purchased from NEB. The RNAP holoenzyme was generated by incubating the core enzyme with a 4‐fold excess of σ factor at room temperature for 20 minutes prior to use. MarA purification was based on that described by Jair *et al. *(1995)*.* T7 Express cells containing pET28a‐MarA were grown to an OD_600_ of 0.8 and expression of MarA was induced with 0.4mM IPTG for 3 h. Cells were harvested by centrifugation and washed with a buffer containing 50mM Tris–HCl (pH 7.5), 1mM EDTA, 1M NaCl before lysis using an AVESTIN EmulsiFlex C3 high pressure motorised homogeniser. Inclusion bodies were collected by centrifugation at 75,000× *g* for 30 minutes and washed with a buffer containing 4M urea, 50mM Tris–HCl (pH 8.5). After recentrifugation, the pellet was solubilised with 50mM Tris–HCl (pH 8.5), 6M guanidinium–HCl. Material remaining in the suspension was removed by centrifugation and His‐tagged MarA was purified from the supernatant by immobilised nickel ion affinity chromatography. The 1ml HisTrap^™^ FF (GE Healthcare) column was equilibrated with Buffer A (1M NaCl, 5mM Tris–HCl (pH 8.5)) and loaded with cell lysate using an ÄKTAprime system (GE Healthcare). Bound protein was eluted using a linear gradient of Buffer B (Buffer A + 1M imidazole). Purified MarA was then transferred into a buffer containing 1M NaCl, 5mM HEPES, 1mM dithiothreitol, 5mM EDTA and 0.1mM Triton X‐100 by dialysis. After concentrating the sample to 1mg/ml with 5,000 MWCO Vivaspin^®^ 20 columns (Sartorius) the peptide bond linking the His‐tag to MarA was cleaved using thrombin sepharose beads (BioVision) for 5 h at room temperature. Beads were removed by centrifugation and the His‐tag was removed by a second round of affinity chromatography. Untagged MarA that did not bind the HisTrap^™^ column was transferred into 1M NaCl, 5mM HEPES, 1mM dithiothreitol, 5mM EDTA, 0.1mM Triton X‐100 and 20% (*v/v*) glycerol by dialysis. Samples were stored at −80 °C until use.

### Electrophoretic mobility shift assays

Assays were performed as described previously (Cosgriff *et al.*, 2010). Briefly, DNA fragments were generated by PCR amplification from an *E. coli* genomic DNA template. Following purification, PCR products were cut with *Hin*dIII and *Eco*RI prior to being end‐labelled with [γ‐32P]‐ATP and polynucleotide kinase. The DNA fragments were incubated with MarA in buffer containing 20 mM Tris pH 7, 10 mM MgCl_2_, 100 mM EDTA and 120 mM KCl. Reactions were analysed by electrophoresis through a 5% polyacrylamide gel. Raw gel images are shown in Fig. S2.

### Assays of *in vitro* transcription

The procedure for *in vitro* transcription was similar to that described (Haycocks *et al.*, 2015) and used the system of Kolb *et al. *(1995). Briefly, a Qiagen Maxiprep kit was utilised to purify pSR plasmid with the required promoter insert. Sixteen microgram per millilitre of DNA template was preincubated with purified MarA in buffer containing 20 mM Tris pH 7.9, 5 mM MgCl_2_, 500 µM DTT, 50 mM KCl, 100 µg ml^‐1^ BSA, 200 µM ATP, 200 µM GTP, 200 µM CTP and 10 µM UTP with 5 µCi [α‐32P]‐UTP. The reaction was started by adding RNAP holoenzyme. Labelled RNA products were analysed on a denaturing polyacrylamide gel. Raw gel images are shown in Fig. S2.

### β‐galactosidase assays

DNA fragments containing the desired derivative of the *ycgZ*‐*ymgABC* regulatory region were cloned in plasmid pRW50 to generate promoter::*lacZ* fusions. The β‐galactosidase levels in lysates of cells carrying these recombinants were measured by the Miller method (Miller, 1972). Activities are the average of three or more independent experiments.

### Congo red binding assays

Bacterial strains were cultured overnight in lysogeny broth (LB) lacking salt (10 g/L of tryptone and 5 g/L of yeast extract). Curli fibres were detected by spotting 5 μl of overnight culture onto LB agar lacking salt and supplemented with 40 µg/ml of Congo red. The agar plates were then incubated at 37°C overnight. The morphology and colour of colonies were recorded by digital photography. The experiments were done multiple times to check that colony phenotypes were reproducible and images shown are representative. The raw image is shown in Fig. S2.

### Crystal violet binding assays

The crystal violet assay described in Baugh *et al. *(2014) was used to quantify biofilm production between bacterial strains. Two independent overnight cultures per strain were diluted in LB to an OD_600_ of 0.1. A 200 μl aliquot was added to a flat‐bottomed 96‐well microtitre plate, with four replicate wells per culture. The plate was incubated at 30°C for 48 h. Wells were washed with water to remove unattached cells and 200 μl of 0.1% w/*v* crystal violet was added for 15 minutes. Wells were then washed with water again to remove unbound crystal violet and 200 μl of 70% ethanol was added to solubilise the retained crystal violet. The A_600_ was then measured using a CLARIOstar plate reader (BMG Labtech) to give a quantitative measure of biofilm formation.

### Bacterial two‐hybrid assays

*In vivo* protein–protein interactions were detected using BacterioMatch II Two‐Hybrid System (Dove and Hochschild, 2004). Interaction of coexpressed hybrid proteins linked to the NTD of lambda cI (from pBT) and to the bacterial RNAP alpha‐NTD (from pTRG) activates *HIS3* gene expression suppressing histidine auxotrophy of the reporter strain (*E. coli* XL1‐Blue MRF’ derivative). The assay was performed according to the instruction manual (Stratagene, Agilent technologies). Cotransformants were obtained on nonselective plates and five independent clones were patched on both, nonselective and selective medium respectively. Growth on selective medium containing the His3 inhibitor 3‐amino‐1,2,3‐triazole (3‐AT) indicates the interaction of the tested hybrid proteins leading to increased expression of *HIS3* gene. Plates were incubated for 24 h at 37°C and for an additional 48 h at 28°C.

### *In vivo* coelution and immunoblot analysis

*In vivo* protein–protein interactions were also analysed using coelution (‘pull‐down’) assays. pBAD33‐6xHis‐*rcsC* (cytosolic part only), pBAD33‐6xHis‐*rcsB*, pBAD‐6xHis‐*ymgA* and pBAD18‐*ymgB*‐Strep were transformed into *E. coli* K‐12 MC4100 containing chromosomally Flag‐tagged *ymgC*, *ymgA* and *ycgZ* respectively. Expression of pBAD‐encoded genes was induced with 0.1% arabinose at OD_600_ of 0.8. Cells were grown overnight at 28°C and cell pellets were lysed using a French press. Cells expressing His‐tagged genes were lysed in ‘His‐lysis buffer’: 50 mM NaH_2_PO_4_, 150 mM NaCl, 20 mM imidazole, pH 8. Strep lysis buffer (100 mM Tris–HCl pH8, 150 mM NaCl, 1 mM EDTA) was used for lysis of cells expressing *ymgB*‐Strep. Ni‐NTA agarose (Qiagen) was used for affinity chromatography of His‐tagged proteins. Strep‐Tactin Sepharose (IBA, Gottingen) was served for affinity purification of YmgB‐Strep. Chromatography columns were washed with the respective lysis buffer and elution was performed using 50 mM NaH_2_PO_4_, 150 mM NaCl, 250 mM imidazole, pH 8 for His‐tagged proteins and buffer E (IBA) for YmgB‐Strep. Copurified Flag‐tagged proteins were detected using monoclonal anti‐FLAG antibody (Sigma) following SDS polyacrylamide gel electrophoresis. To detect protein–protein interactions between YmgB‐Strep and either 6xHis‐RcsC (cytosolic part) or 6xHis‐RcsB, respectively, pBAD33‐6xHis‐*rcsC* or pBAD33‐6xHis‐*rcsB* were cotransformed and expressed with pBAD18‐*ymgB*‐Strep in MC4100. The cells were grown and treated as described above. His‐tagged proteins were purified using Ni‐NTA. Coeluted YmgB‐Strep was detected using anti‐Strep‐Tactin antibody (IBA).

## Data Availability

All data are available within the manuscript figures or within the cited references.
