# Supplementary material for: The Escherichia coli MarA protein regulates the ycgZ‐ymgABC operon to inhibit biofilm formation
Source: Mol Microbiol. 2019 Sep 29;112(5):1609–25. doi: 10.1111/mmi.14386 (PMC6900184; doi:10.1111/mmi.14386)
Supplement: Supplementary file 1 [file MMI-112-1609-s001.pdf]

Supplementary Information

The *Escherichia coli* MarA protein regulates the *ycgZ-ymgABC* operon to inhibit biofilm formation

Rachel A. Kettles[1], Natalia Tschowri[2], Kevin J. Lyons[1], Prateek Sharma[1], Regine Hengge[2], Mark A. Webber[3], David C. Grainger[1]*

*for correspondence

email: d.grainger@bham.ac.uk

Tel +44 (0)121 414 5437

[1]Institute of Microbiology and Infection, School of Biosciences, University of Birmingham, Edgbaston, Birmingham B15 2TT, United Kingdom

[2]Institut für Biologie/Mikrobiologie, Humboldt-Universität zu Berlin, 10115 Berlin, Germany

[3]Quadram Institute Bioscience, Norwich Research Park, Norwich NR4 7UQ, United Kingdom

Figure S1

ycgZ.1

ATATGCATTAGCACTAATTGCAAAAAATTAATTTATCATTCTGTACACATATTTCGT
ACAAGTTTGCTATTGTTACTTCACTTAACATTGATTAACATTTTTAACAGAGGCGTAGCATG

ycgZ.1 inv

ATATGCATTAAAAACGTTAATCACGAATTAATTTATCATTCTGTACACATATTTCGT
ACAAGTTTGCTATTGTTACTTCACTTAACATTGATTAACATTTTTAACAGAGGCGTAGCATG

ycgZ.1 Δ1

ATATGCATTAGCACTAATTGCAAAAATTAATTTATCATTCTGTACACATATTTCGTA
CAAGTTTGCTATTGTTACTTCACTTAACATTGATTAACATTTTTAACAGAGGCGTAGCATG

ycgZ.1 Δ5

ATATGCATTAGCACTAATTGCAAAAATTTATCATTCTGTACACATATTTCGTACAA
GTTTGCTATTGTTACTTCACTTAACATTGATTAACATTTTTAACAGAGGCGTAGCATG

ycgZ.1 Δ10

ATATGCATTAGCACTAATTGCAAAATCATTCTGTACACATATTTCGTACAAGTTTG
CTATTGTTACTTCACTTAACATTGATTAACATTTTTAACAGAGGCGTAGCATG

ycgZ.1 UP1

ATATGCATTAGCACTAATTGCAAAA*C*AATTAATTTATCATTCTGTACACATATTTCGT
ACAAGTTTGCTATTGTTACTTCACTTAACATTGATTAACATTTTTAACAGAGGCGTAGCATG

ycgZ.1 UP5

ATATGCATTAGCACTAATTGCAAAA*CGATC*AATTAATTTATCATTCTGTACACATATTTCGT
ACAAGTTTGCTATTGTTACTTCACTTAACATTGATTAACATTTTTAACAGAGGCGTAGCATG

ycgZ.1 UP10

ATATGCATTAGCACTAATTGCAAAA*TCTGACGATC*AATTAATTTATCATTCTGTACACATATTT
CGTACAAGTTTGCTATTGTTACTTCACTTAACATTGATTAACATTTTTAACAGAGGCGTAGCATG

Figure S2

Figure 2a

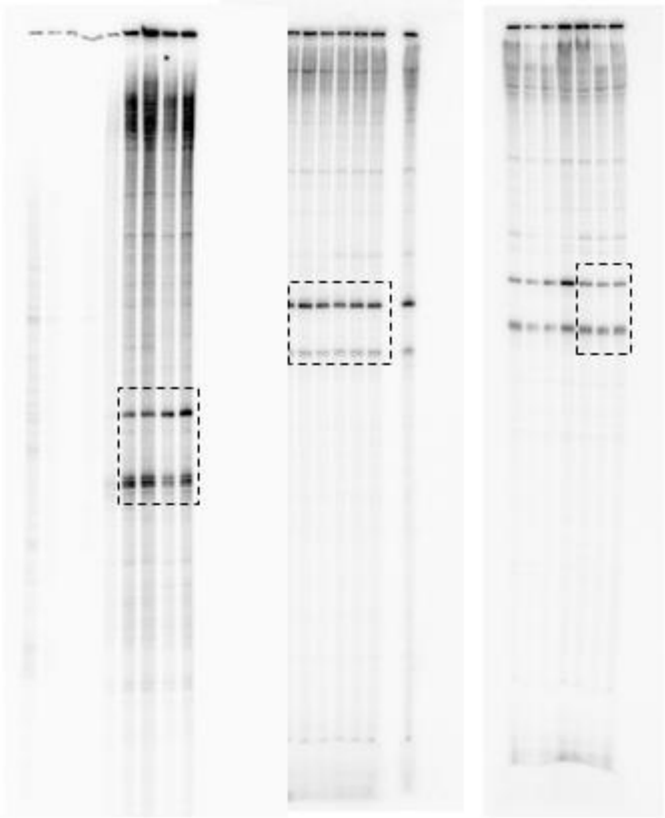

Figure 1b

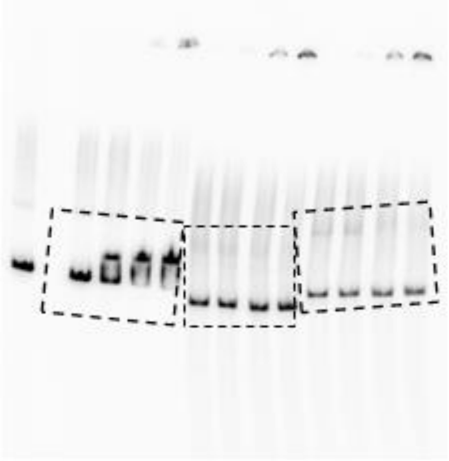

Figure 2b

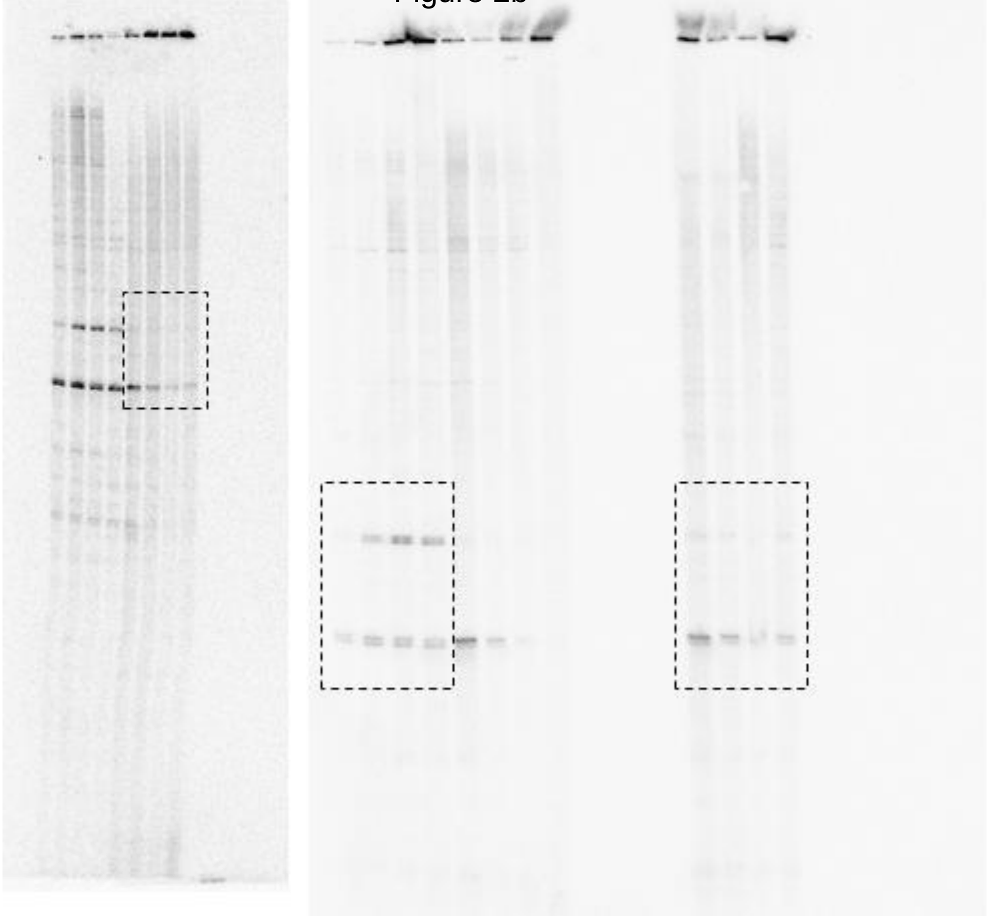

Figure 3b

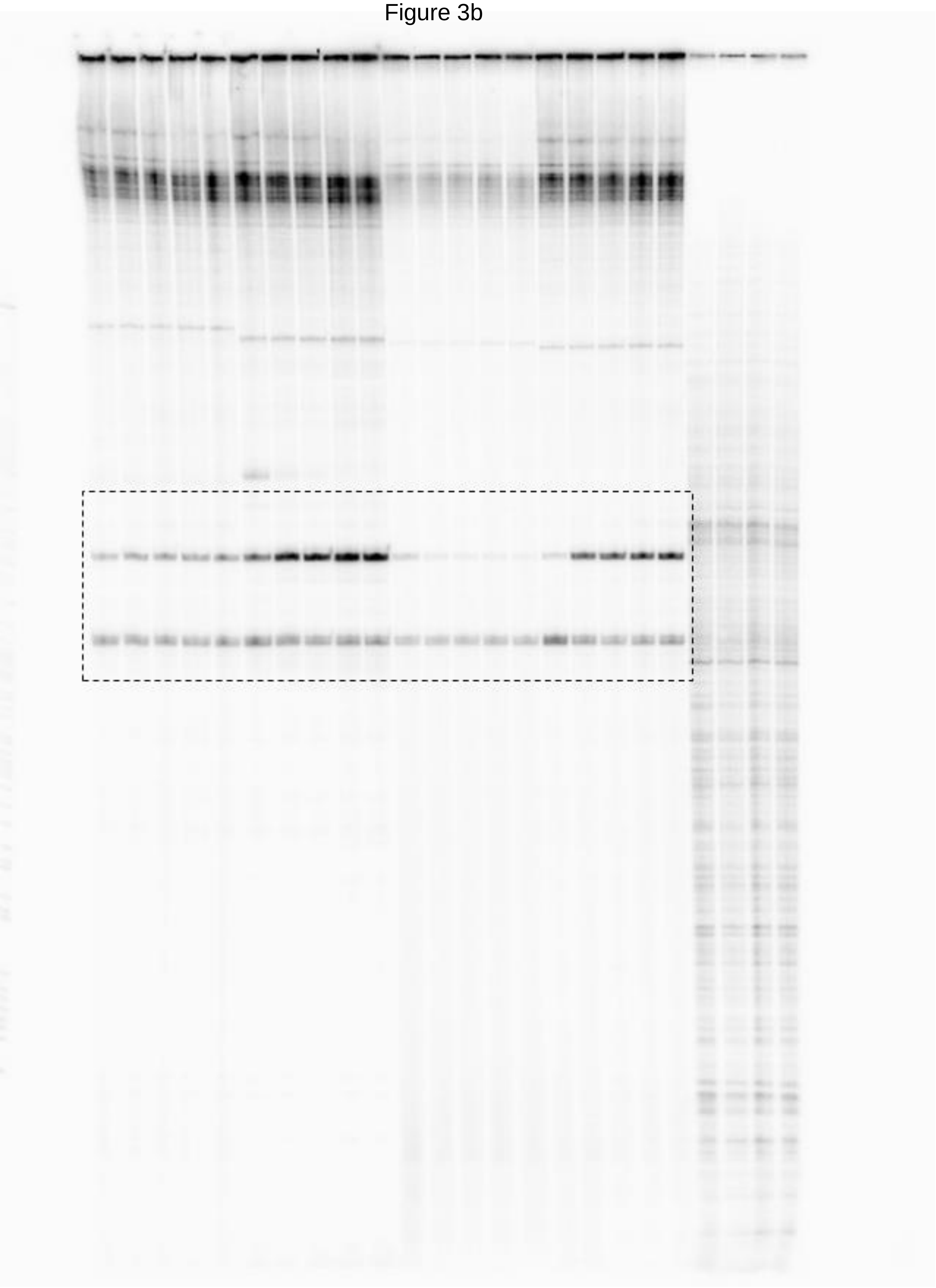

Figure 3b

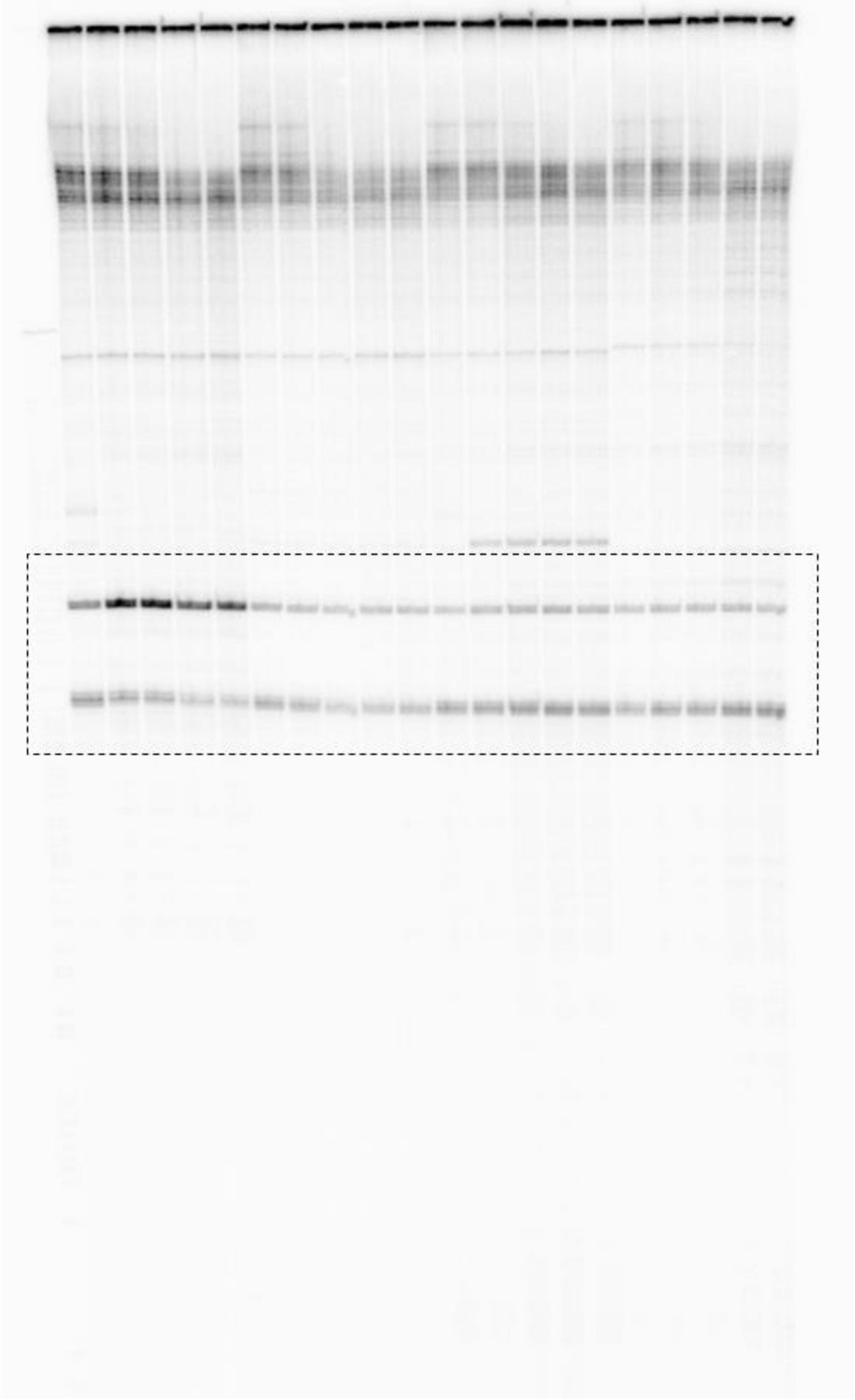

Figure 3c

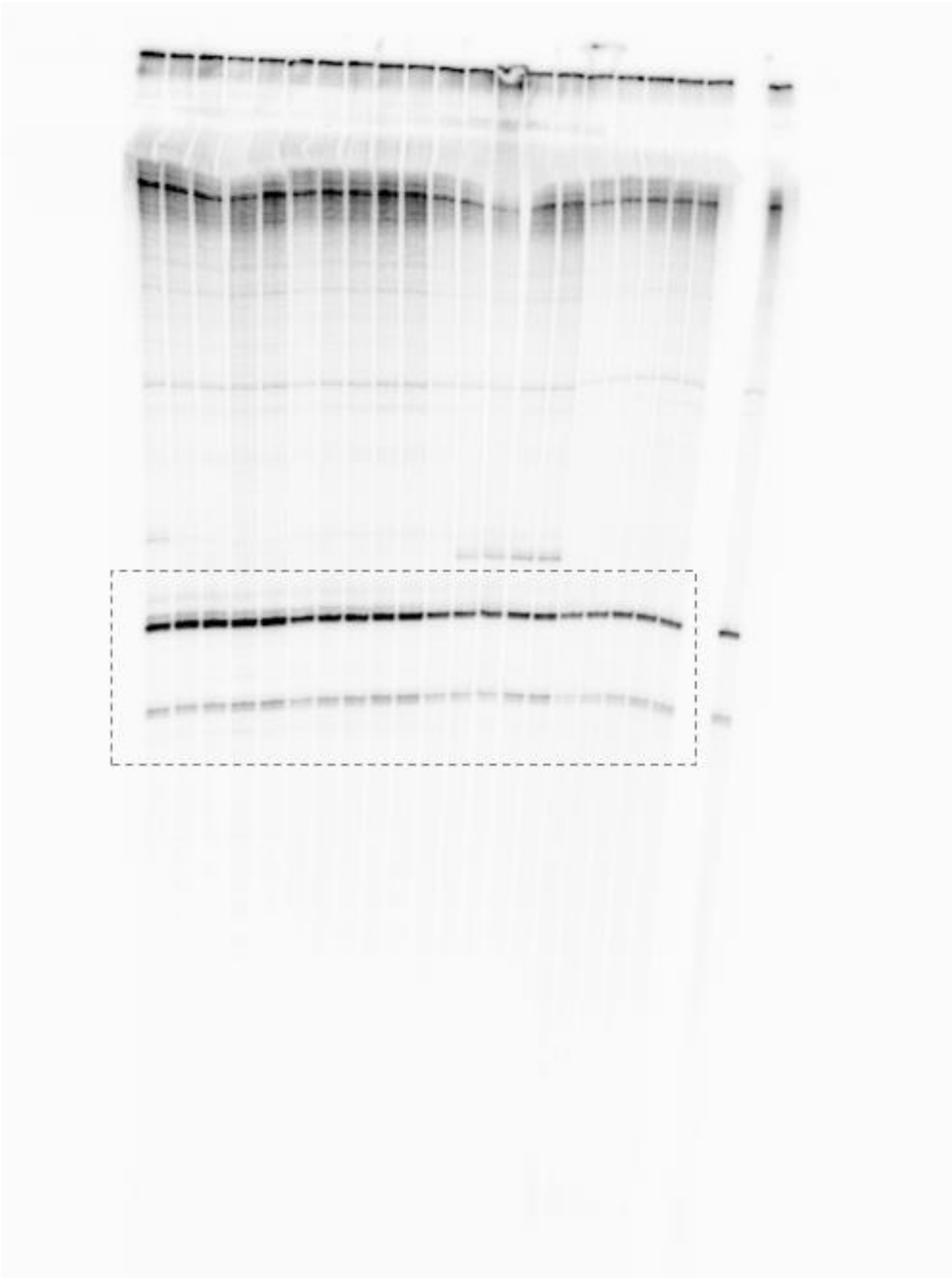

Figure 3c

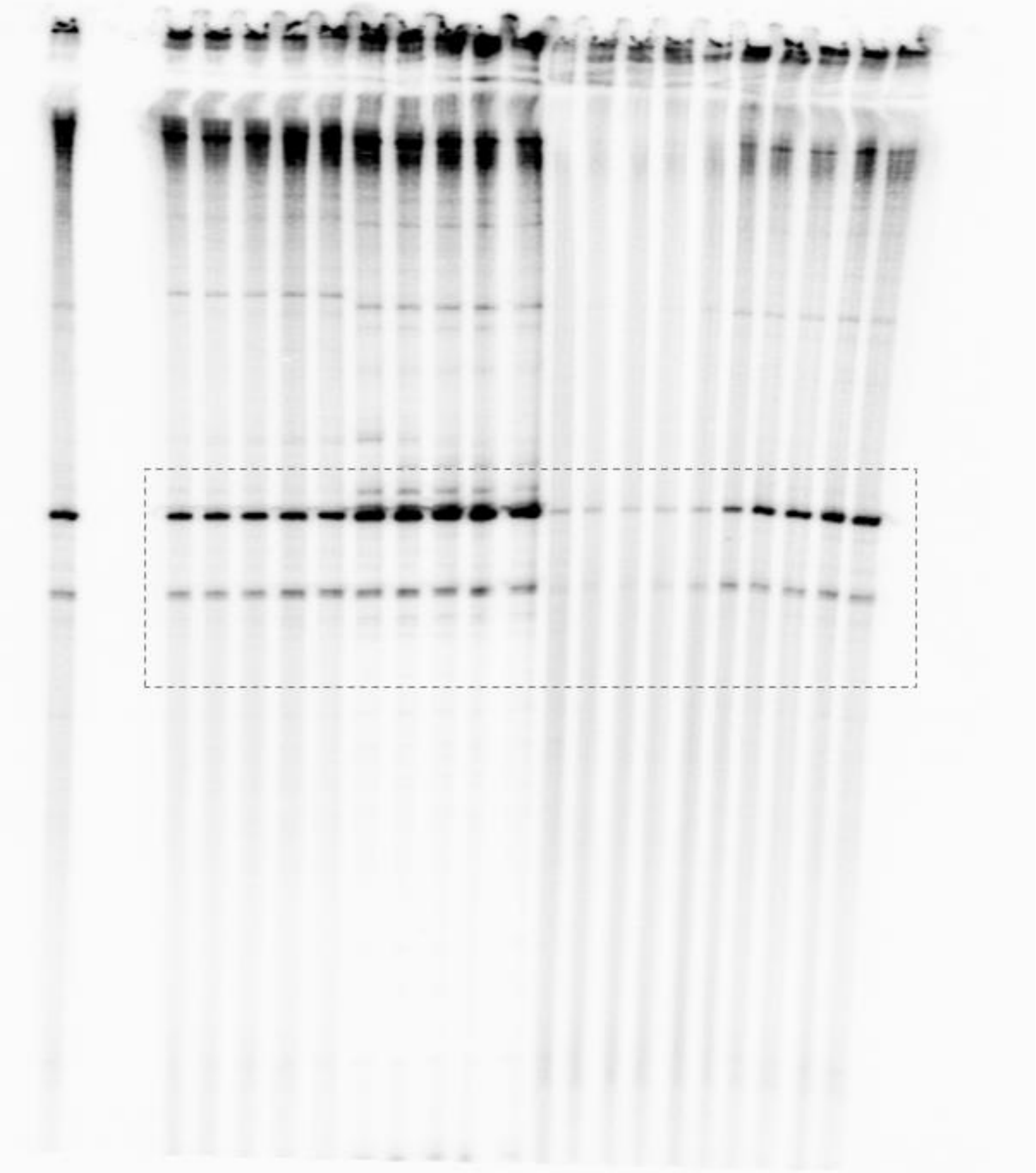

Figure 5

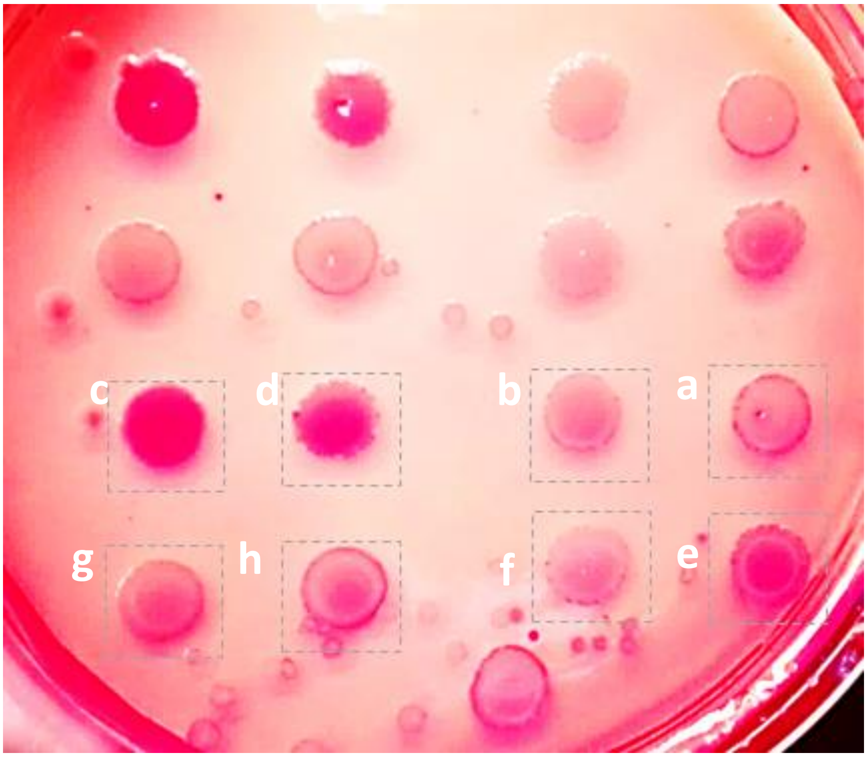

Repeat spotting of below two rows in the same order

Data shown in Figure 5.
