# Supplementary material for: The Escherichia coli MarA protein regulates the ycgZ‐ymgABC operon to inhibit biofilm formation
Source: Mol Microbiol. 2019 Sep 29;112(5):1609–25. doi: 10.1111/mmi.14386 (PMC6900184; doi:10.1111/mmi.14386)
Supplement: Supplementary file 2 [file MMI-112-1609-s002.docx]

**SUPPLEMENTARY FIGURE LEGENDS**

**Figure S1: Sequences of *ycgZ*.1 derivatives.** The marbox is green and promoter elements are underlined. The site of deletions is shown by an inverted triangle. Inserted sequences are in italic.

**Figure S2: Raw images.**
