# Supplementary material for: The Escherichia coli MarA protein regulates the ycgZ‐ymgABC operon to inhibit biofilm formation
Source: Mol Microbiol. 2019 Sep 29;112(5):1609–25. doi: 10.1111/mmi.14386 (PMC6900184; doi:10.1111/mmi.14386)
Supplement: Supplementary file 3 [file MMI-112-1609-s003.docx]

**Table S1: Oligonucleotides**

**Name Sequence Description**

***Oligonucleotides***

*ycgZ*.1-F 5'-gctgctgaattcatatgcattagcactaattgca-3' Used with *ycgZ*-R to make the

*ycgZ*.1 fragment, or with *ymgC-E* to make the fragment comprising the *ycgZ*-*ymgABC* genes under the control of *ycgZ*.1

*ycgZ*.2-F 5'-ggtgtcgaattcaatttatcattctgtacacatatt Used with *ycgZ-R* to make the

tcg-3' *ycgZ*.2 fragment, and with *ymgC-E*

to make the fragment comprising the

*ycgZ-ymgABC* genes under the

control of ycgZ.2.

ycgZ.1^m^-F 5'-gctgctgaattcatatgcattacgtctaattcga Used with *ycgZ-*R to make the

aaaaattaatttatcattctgtacacatatttcg-3' *ycgZ*.1^m^ fragment, and with *ymgC*-*E*

to make the fragment comprising

*ycgZ-ymgABC* genes under the

control of *ycgZ*.1^m^

*ycgZ*.1-inv-F 5'-gctgctgaattcatatgcattaaaaacgttaatc Used with *ycgZ-*R to make the

acgaattaatttatcattctgtacac-3' *ycgZ*.1inv fragment.

*ycgZ*.1-Δ1-F 5'-gctgctgaattcatatgcattagcactaattgca Used with *ycgZ-*R to make the

aaaattaatttatcattctgtacac-3' *ycgZ*.1 Δ1 fragment.

*ycgZ*.1-Δ5-F 5'-gctgctgaattcatatgcattagcactaattgca Used with *ycgZ-*R to make the

aaaatttatcattctgtacac-3' *ycgZ*.1 Δ5 fragment.

*ycgZ*.1-Δ10-F 5'-gctgctgaattcatatgcattagcactaattgca Used with *ycgZ-*R to make the

aaatcattctgtacacatatttcg-3' *ycgZ*.1 Δ10 fragment.

*ycgZ*.1-U1-F 5'-gctgctgaattcatatgcattagcactaattgca Used with *ycgZ-*R to make the

aaacaattaatttatcattctgtacac-3' *ycgZ*.1 U1fragment.

*ycgZ*.1-U5-F 5'-gctgctgaattcatatgcattagcactaattgca Used with *ycgZ-*R to make the

aaacgatcaattaatttatcattctgtacac-3' *ycgZ*.1 U5 fragment.

*ycgZ*.1-U10-F 5'-gctgctgaattcatatgcattagcactaattgca Used with *ycgZ-*R to make the

aaatctgacgatcaattaatttatcattctgtacac-3' *ycgZ*.1 U10 fragment.

*ycgZ*-R 5'-agtagtaagcttcatgctacgcctctgtta-3' Used in conjunction with *ycgZ*.1-F,

*ycgZ*.2-F, or *ycgZ*.1^m^-F to generate

*ycgZ-ymgABC* promoter

derivatives.

*ymgC-E*  5'-gcattggacgtcctaagagagcacggattc-3' Used in conjunction with *ycgZ*.1-F,

*ycgZ*.2-F, and *ycgZ.1*^m^-F to generate

*ycgZ-ymgABC* and promoter derivatives F cloning in pBR322Δbla

*ycgZ*-*ymgABC* 5'-gctgctgaattcttcacttaacattgattaaca F deletion of the chromosomal

-GD-F tttttaacagaggcgtagcgaccggtcaattggc *ycgZ-ymgABC* operon by gene

tggag-3' doctoring. *Eco*RI and *Hin*dIII sites

are incorporated F cloning into

pDOC-C.

*ycgZ-ymgABC* 5'-agtagtaagcttgtgatacagctgatgtttatt Gene doctoring primer F deletion

-GD-R ctaaaaccttactcaagttaatatcctccttagtt of the chromosomal *ycgZ-ymgABC*

cc-3' operon. Used in conjunction with

*ycgZ-ymgABC*-GD-F.

MarA-OE-F 5'-cccaattccatatgtccagacgcaatactgacgc-3' Used with MarA-OE-R to generate a

fragment containing the *marA* gene F cloning in pET28a.

MarA-OE-R 5'-ggcggatccctacgacttatcactgccagtacc-3' Used with MarA-OE-F.

Δ*bla*-F 5'-gcatctattaatccagcactaactacgatgcgcag Used with Δ*bla*-R to make linker F

cgataagcaggtaggtcaacgtgcgatgcgtcattc replacing the pBR322 *bla* gene.

ggattgcgatttagc-3'

Δ*bla*-R 5'-gcatgagacgtcaagtaacgatgctctgactcgaa Used with Δ*bla*-F.

gatagacttgtgttctctaagctaaatcgcaatccga

atgacgcatcgcac-3'

MarAW19A-F 5'-cccaattccatatgacgatgtccagacgcaatactg Used to introduce W19A mutation

acgctattaccattcatagcattttggacgcgatcgag

gacaacctggaat-3'

YcgZSUB11-F 5'-cagaaacattataatgcactaattgggttgcttttt Generation of ycgZ::Flag

gaagactacaaagaccatgacgg-3'

YcgZSUB11-R 5'-catagctaacctgttaaataatcaggctgttgcatt Generation of ycgZ::Flag

acatatgaatatcctccttag-3'

YmgASUB11F 5'-aagtatgttcaatggtaagaaaataaacagaataca Generation of *ymgA*::Flag

tgactacaaagaccatgacgg-3'

YmgASUB11R 5'-caagcatggtaacctctcatcttacttatgaaattt Generation of *ymgA*::Flag

tacatatgaatatcctccttag-3'

YmgCSUB11F 5'-cggatatcattattaatgacagggaatccgtgctct Generation of *ymgC*::Flag

ctgactacaaagaccatgacgg-3'

YmgCSUB11R 5'-cagctgatgtttattctaaaaccttactcaagttct Generation of *ymgC*::Flag

acatatgaatatcctccttag-3'

His6-*rcsB*-F 5'-cggaattcaggaggtactgagatgcatcaccatcat Cloning of His-*rcsB* in caccacggcgccgagaacaatatgaacgtaattattgc pBAD33

cg-3'

His6-*rcsB*-R 5'-cgcaagctttcagtctttatctgccggac-3' Cloning of His-*rcsB* in

pBAD33

His6-*rcsC*-F 5'-cggaattcaggaggtactgagatgcatcaccatcat Cloning of His-*rcsC* into caccacggcgccgaggaactggcgcatacctatctc-3' pBAD33

His6-*rcsC*-R 5'-cgcaagctttcacgaatcccgcgatttcctgac-3' Cloning of His-*rcsC* into

pBAD33

His6-*ymgA*-F 5'-cggagctcaggaggactgagatggctagccatcacc Cloning of His-*ymgA* into atcatcaccacggcgccaagacatctgataatgaacgt pBAD33

at-3'

His6-*ymgA*-R 5'-cgtaagctttcaatgtattctgtttattttctacc Cloning of His-*ymgA* into

attg-3' pBAD18

*ymgB*-Strep-F 5'-cggaattcaggaggtactgagatgcttgaagatac Cloning of *ymgB*-Strep into

tacaattc-3' pBAD18

*ymgB*-Strep-R 5'-cgtaagctttcattatttttcgaactgcgggtggc Cloning of *ymgB*-Strep into

tccacatatcatcagctgtgtatcgc-3' pBAD18

*rcsC*-pTRG-F 5'-cgcgaattcaggaactggcgcatacctatctc-3' Cloning of *rcsC* into pTRG

*rcsC*-pTRG-R 5'-cgcctcgagtcacgaatcccgcgatttcctgac-3' Cloning of *rcsC* into pTRG

*rcsC*HKpTRGF 5'-cgcgaattcaggaactggcgcataccta tctc-3' Cloning of *rcsC* histidine kinase

domain into pTRG

*rcsC*HKpTRGR 5'-cgcctcgagtcaccagcagcgtttaccac tcaa-3' Cloning of *rcsC* histidine kinase

domain into pTRG

*rcsC*ABLTRGF 5'-cgcgaattcaggcgtcgctctgtcagttcctg-3' Cloning of *rcsC* alpha beta loop

domain into pTRG

*rcsC*ABLTRGR 5'-cgcctcgagtcagtcattatcgctgaccgctttg Cloning of *rcsC* alpha beta loop

tc-3' domain into pTRG

*rcsC*PRpTRGF 5'-cgcgaattcagctggcagatcagttgggatcg-3' Cloning of *rcsC* phosphor receiver

domain into pTRG

*rcsC*PRpTRGR 5'-cgcctcgagtcacgaatcccgcgatttcctgac-3' Cloning of *rcsC* phosphor receiver

domain into pTRG

*rcsD*-pTRG-F 5'-cggaattcagcgccatttctccagccgc-3' Cloning of *rcsD* into pTRG

*rcsD*-pTRG-R 5'-cgctcgagtcacagcaagctcttgacataac-3' Cloning of *rcsD* into pTRG

*rcsB*-pTRG-F 5'-cggaattcagaacaatatgaacgtaattattgc-3' Cloning of *rcsB* into pTRG

*rcsB*-pTRG-R 5'-cgcaagctttcacgaatcccgcgatttcctgac-3' Cloning of *rcsB* into pTRG

*ymgA*-pTRG-F 5'-cgcgaattcagaagacatctgataatgaac gtat-3' Cloning of *ymgA* into pTRG

*ymgA*-pTRG-R 5'-cgcctcgagtcaatgtattctgtttattttct tac Cloning of *ymgA* into pTRG

c-3'

*ymgB*-pTRG-F 5'-cggaattcagcttgaagatactacaattc-3' Cloning of *ymgB* into pTRG

*ymgB*-pTRG-R 5'-cgcctcgagtcacatatcatcagctgtgtatcgc-3' Cloning of *ymgB* into pTRG

*ymgC*-pTRG-F 5'-cgcgaattcagaataattcaatcccagagag-3' Cloning of *ymgC* into pTRG

*ymgC*-pTRG-R 5'-cgcactagttcaagagagcacggattccctgtc-3' Cloning of *ymgC* into pTRG

*rcsD*-pBT-F 5'-cggaattcccgccatttctccagccgc-3' Cloning of *rcsD* into pBT

*rcsD*-pBT-R 5'-cgctcgagtcacagcaagctcttgacataac-3' Cloning of *rcsD* into pBT

*ycgZ*-pBT-F 5'-cgcgaattcccatcaaaattcagtgactttag-3' Cloning of *ycgZ* into pBT

*ycgZ*-pBT-R 5'-cgcctcgagtcattcaaaaagcaacccaattag-3' Cloning of *ycgZ* into pBT

*ymgA*-pBT-F 5'-cgcgaattccaagacatctgataatgaacgtat-3' Cloning of *ymgA* into pBT

*ymgA*-pBT-R 5'-cgcctcgagtcaatgtattctgtttattttct ta Cloning of *ymgA* into pBT

cc-3'

*ymgB*-pBT-F 5'-cgcgaattcccttgaagatactacaattc-3' Cloning of *ymgB* into pBT

*ymgB*-pBT-R 5'-cgcctcgagtcacatatcatcagctgtgta tcgc-3' Cloning of *ymgB* into pBT

*ymgC*-pBT-F 5'-cgcgaattccaataattcaatcccagagag-3' Cloning of *ymgC* into pBT

*ymgC*-pBT-R 5'-cgcagatcttcaagagagcacggattccctgtc-3' Cloning of *ymgC* into pBT
